# The Non-Flagellar Type III Secretion System Evolved from the Bacterial Flagellum and Diversified into Host-Cell Adapted Systems

**DOI:** 10.1371/journal.pgen.1002983

**Published:** 2012-09-27

**Authors:** Sophie S. Abby, Eduardo P. C. Rocha

**Affiliations:** 1 Institut Pasteur, Microbial Evolutionary Genomics, Département Génomes et Génétique, Paris, France; 2 CNRS, UMR3525, Paris, France; Environmental Research Institute, University College Cork, Ireland

## Abstract

Type 3 secretion systems (T3SSs) are essential components of two complex bacterial machineries: the flagellum, which drives cell motility, and the non-flagellar T3SS (NF-T3SS), which delivers effectors into eukaryotic cells. Yet the origin, specialization, and diversification of these machineries remained unclear. We developed computational tools to identify homologous components of the two systems and to discriminate between them. Our analysis of >1,000 genomes identified 921 T3SSs, including 222 NF-T3SSs. Phylogenomic and comparative analyses of these systems argue that the NF-T3SS arose from an exaptation of the flagellum, i.e. the recruitment of part of the flagellum structure for the evolution of the new protein delivery function. This reconstructed chronology of the exaptation process proceeded in at least two steps. An intermediate ancestral form of NF-T3SS, whose descendants still exist in Myxococcales, lacked elements that are essential for motility and included a subset of NF-T3SS features. We argue that this ancestral version was involved in protein translocation. A second major step in the evolution of NF-T3SSs occurred *via* recruitment of secretins to the NF-T3SS, an event that occurred at least three times from different systems. In rhizobiales, a partial homologous gene replacement of the secretin resulted in two genes of complementary function. Acquisition of a secretin was followed by the rapid adaptation of the resulting NF-T3SSs to multiple, distinct eukaryotic cell envelopes where they became key in parasitic and mutualistic associations between prokaryotes and eukaryotes. Our work elucidates major steps of the evolutionary scenario leading to extant NF-T3SSs. It demonstrates how molecular evolution can convert one complex molecular machine into a second, equally complex machine by successive deletions, innovations, and recruitment from other molecular systems.

## Author Summary

Most motile bacteria use a flagellum to move. The extracellular components of flagella are secreted by their own Type 3 Secretion System (T3SS). The non-flagellar T3SS (NF-T3SS), also named injectisome, includes many proteins that are homologous to flagellar components. NF-T3SSs are employed by many plant and animal pathogens to deliver effectors to host cells, including toxins. NF-T3SSs are complex protein machineries with >15 components that connect bacterial cell envelopes to eukaryotic cell membranes, including the intervening extracellular space. In this study, we designed computational tools to distinguish flagella and NF-T3SSs from other bacterial protein sequences. We show that NF-T3SSs evolved from the flagellum by a series of genetic deletions, innovations, and recruitments of components from other cellular structures. Our evolutionary analysis suggests that NF-T3SSs then quickly adapted to different eukaryotic cells while maintaining a core structure that remains highly similar to the flagellum. This is an example of evolutionary tinkering where a complex structure arises by exaptation, the recruitment of elements that evolved initially for other functions in other cellular structures.

## Introduction

Microbial protein secretion facilitates environmental exploitation and manipulation [1], [2]. The most frequent bacterial motility machinery, the flagellum, uses a type 3 secretion system (T3SS) to secrete its extracellular components. The non-flagellar type 3 protein secretion system (NF-T3SS), often named injectisome, is homologous to the flagellum and also acts as a T3SS. This system is used to secrete its extracellular components and to deliver effectors into host cells. The NF-T3SS is an important virulence factor for animal pathogens within *Salmonella*, *Escherichia*, *Chlamydia* and *Yersinia* and plant pathogens within *Xanthomonas*, *Ralstonia* or *Burkholderia* (for reviews see [3]–[8]). Some genomes encode several NF-T3SSs. In *Burkholderia pseudomallei* discrete NF-T3SSs are involved in animal and plant pathogenesis [9]–[11], and in *Salmonella enterica* two NF-T3SSs are used in different phases of infection [12]. NF-T3SSs are exposed at the cell surface and are therefore targeted by the immune response. As a result, NF-T3SSs are being studied as targets for vaccines, e.g. for protection against *Shigella* [13], and for new antibacterial drugs [14]. They have also been used to deliver vaccine antigens to the cytosol of eukaryotic cells [15], [16]. NF-T3SSs have been most thoroughly studied for their role in antagonistic associations between pathogens and their hosts, but they also play an important role in mutualistic associations between bacteria and insects [17], plants [18], or fungi [19]. Phylogenetic evidence indicates that NF-T3SSs originated before most multicellular eukaryotes, possibly to favor interactions with early unicellular eukaryotes [20].

Each model NF-T3SS has its unique nomenclature in the literature. Hence, we use a unifying nomenclature throughout this publication [3], naming the NF-T3SS core components with the prefix *sct* for secretion and cellular translocation, followed by the suffixes used in the *Yersinia* Ysc system (see Table S1 for correspondence with other systems). When no unique name has been proposed we use the name of the *Yersinia* system by default, unless specifically specified. To avoid ambiguity, we follow Desvaux *et al.* [21] and use “translocation” for transport through a lipid bilayer and “secretion” for transport from the interior to the exterior of the cell. We use the term “delivery” to refer to the active transport from the interior of one cell to the cytosol of a second cell.

**Figure 1:**
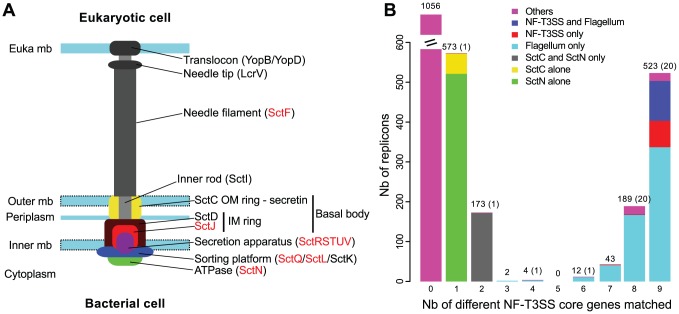
Identification of T3SS core genes in complete genomes. (A) Representation of the proposed organization of a NF-T3SS system, inspired from the *Salmonella* system [139]. For consistency with the main text, nomenclature follows that of *Yersinia* for some genes (see Table S1 for equivalence). Genes in red have homologs in the flagellum (main text, Table S1). “Euka” stands for eukaryotic, “mb” stands for membrane, “IM” for inner membrane, and “OM” for outer membrane. (B) Distribution of the number of different protein profiles of the 9 core genes of NF-T3SS matched per replicon (i-evalue<10^−3^). Bar colors discriminate between systems in flagella or NF-T3SSs and other systems including only a secretin (SctC) and/or the ATPase (SctN). Numbers of replicons are indicated above the corresponding bars, with the number of uncharacterized cases (“Others”, colored in magenta in bars) in parenthesis if different from the total. “Nb” is an abbreviation for “Number”.

The NF-T3SS is a complex protein structure that spans the cytoplasmic and outer membranes of bacteria and the cell envelope of the eukaryotic host to deliver effectors directly into its cytosol (Figure 1A). The basal body of the NF-T3SS provides a structural basis for the secretion machinery involved in protein delivery. The outer membrane component of the basal body is formed by a homo-polymeric ring of secretins (SctC) [22], [23], whose inactivation leads to the accumulation of effectors in the periplasm [24]. This protein is part of a large family of pore-forming secretins, also found in Type IV pili (T4P), Type II Secretion Systems (T2SS), Flp pili encoded by the tight adherence (Tad) system and the extrusion machinery of filamentous phages [25]. At the inner-membrane the basal body inner ring is composed of SctJ and SctD [22], [26]. The needle of the NF-T3SS is connected to the basal body at the inner membrane and extends outward from the cell [27]. In *Salmonella*, *Shigella* and *Yersinia* the needle is made of a major subunit (SctF) [26], [28], [29]. Phytopathogens do not have a needle but a flexible pilus-like structure encoded by HrpA [30], [31]. HrpA shares several traits with SctF and its pilus presumably represents an adaptation of the NF-T3SS to the thick plant cell wall [32]. The needle is capped by a tip (LcrV) involved in regulating secretion and in positioning the translocation pore in the host cell membrane [33], [34]. When the system is in contact with a target cell and delivery is activated, the translocon (YopB and YopD) acts as a pore-former in the eukaryotic membrane [35]. A few highly conserved proteins underneath the basal body are essential for the function of the NF-T3SS. One is a member of the F-/V- ATPase family (SctN), with homologs in flagella and F_0_F_1_ proton-translocating ATPases [36]–[39]. This ATPase functions in the recognition and unfolding of secreted proteins and possibly participates in energizing the process of secretion [40]–[43]. In *S. enterica* Typhimurium, a protein similar to the flagellum C-ring “sorting” platform (SctQ) was suggested to orchestrate the order of protein secretion in interaction with SctK and SctL [44]. The remaining highly conserved proteins (SctRSTUV) form the secretion apparatus. Their functions are poorly understood but they are thought to include substrate selection and molecular switching between modes of secretion [45], [46]. In *Salmonella, Shigella*, and *Yersinia*, another protein of importance, SctP, controls the needle-length during its assembly [26], [47], [48]. In *Yersinia*, SctP and SctU are involved in substrate-switching [45], [49], possibly by regulating the export of the inner rod protein (SctI) [49].

Two models were proposed for the initial stages of NF-T3SS assembly. In the outside-in model, inferred primarily from *Yersinia* data [50], [51], the initial assembly of the secretin rings (SctC) at the outer membrane favors assembly of the inner membrane rings by interacting with SctD. The secretion apparatus then assembles independently of the basal body. Together, these two structures recruit SctJ at the inner membrane. In the inside-out model, inferred primarily from *Salmonella* data [52], assembly starts with the secretion apparatus, which provides a platform stabilizing the inner ring association between SctJ and SctD. The interaction of SctD and the secretin stabilizes the outer membrane rings. The SctQ C-ring and SctN ATPase are then recruited to the complex in both models. The general secretory pathway translocates the non-cytoplasmic components of this initial complex but once the ATPase is in place, the remaining components, including the inner rod (SctI), the needle filament (SctF) and the tip (LcrV), are secreted by the assembling NF-T3SS itself [53]. Upon contact with a host-cell membrane, the NF-T3SS initially secretes the translocators and then the effectors. The processes of NF-T3SS assembly and secretion of translocators and effectors involve a series of switches to control the timing, order and number of secreted proteins [54]. The flagellum assembly pathway shares many of these organizing principles, including switches of secretion substrates as the appendage forms [55], [56], and inside-out assembly [57]. Interestingly, it is not yet clear if NF-T3SS effectors are delivered through the central channel of the needle/pilus structures, as suggested by the designation “injectisome”. It is possible that the needle is only a sensor and regulator used to establish tight cell contacts and trigger the extracellular secretion of effectors [58], [59] for subsequent translocation to the eukaryotic cell [60].

Experimentally studied NF-T3SSs include between 15 and 25 proteins, of which nine are ubiquitous core proteins (SctCJNQRSTUV) and four other (SctDFLP) are present in most systems (Figure 1A, Table S1) [3], [6], [8], [61]. Phylogenetic analyses of the ubiquitous proteins led to the identification of seven different NF-T3SS types [62], each including one or a few different model systems: SPI1 includes an NF-T3SS from the *S. enterica* Pathogenicity Island 1 and from *Shigella* [27], [63], SPI2 is from a second locus in *S. enterica* [64], [65], Ysc from *Yersinia* [66], Chlamy from *Chlamydia trachomatis* [67], Rhizo from *Rhizobium* sp. strain NGR234 [18], Hrp1 from *Pseudomonas syringae* [68] and Hrp2 from *Ralstonia solanacearum* [69]. Interestingly, this categorization distinguished systems involved in bacterial interactions with animals and protozoa (SPI1, SPI2, Ysc, Chlamy systems) from systems involved in interactions with plants and fungi (Hrp1, Hrp2, Rhizo systems). A number of these NF-T3SS proteins are homologous to the flagellum [70], clearly showing common ancestry of the two structures: of 13 (nearly) ubiquitous proteins in the NF-T3SS, nine have clear homologs in the flagellum (SctJNQRSTUV/L), and two, SctP and SctF, have functional and structural counterparts [71]. SctP proteins play a central role, thought to be analogous to that of the flagellar protein FliK [26], and they are interchangeable between *Shigella* and *Salmonella* SPI1 systems [47]. Unfortunately, they display very weak sequence similarity between closely related bacteria [47], which precludes an evolutionary analysis by sequence similarity or phylogenetic methods. Similar problems arise when analyzing the chaperones of NF-T3SS, which are essential for needle complex formation and secretion, but are too diverse between NF-T3SSs of different families to allow comprehensive evolutionary studies. It is noteworthy that the flagellum outer membrane ring (L-ring) is formed with a lipoprotein unrelated to the secretin (FlgH) [72].

The trees of concatenated sequences of the flagellar components are approximately congruent with a tree of concatenated universal protein sequences [73], suggesting relatively few cases of horizontal transfer [74]. On the other hand, the NF-T3SS has been extensively transferred among Proteobacteria [65], [75]–[77], albeit not among Chlamydiales [78]. When their genes were horizontally transferred, the imports seem to have included the entire set of genes in a single event for both flagellum and NF-T3SS [3], [79], [80]. A decade ago, several studies indicated one single phylogenetic split between the flagellum and the NF-T3SS [75], [79], [81], [82]. This is compatible with three different evolutionary scenarios. The two elements might have independent origins from an ancestral system, or one system might have adapted pre-existing structures from the other system for a new function [61], a process referred to as “exaptation” [83]. Understanding the details of the exaptation process requires an understanding of the direction of the evolutionary events. Current sequence databanks cover a much larger fraction of the prokaryotic world than ten years ago. Phylogenetic methods for dealing with multi-protein complexes have also been improved [84], [85], but these newer approaches have not yet been applied to infer the evolutionary history of T3SSs. The ongoing explosion of partially assembled genomes and metagenomes would also benefit from new tools for the detection and analysis of T3SSs from partial data. We have therefore produced such tools and applied them to genome data in order to determine the evolutionary origins and patterns of diversification of T3SSs.

## Results

### Detection of T3SS components and discrimination from flagellar components

We retrieved sequences corresponding to a diverse set of known flagella and NF-T3SSs from genome databanks. We focused initially on 12 gene families (Table S1), eight with homologs among flagella and NF-T3SSs (the T3SS core genes: *sctJNQRSTUV*), one family specific to NF-T3SSs (the secretin *sctC*) and three specific to flagella (genes encoding rod components: *flgB*, *flgC* and *fliE)*. We searched for homologs of these genes, made multiple alignments, manually corrected them and built protein profiles (Materials and Methods). We queried the proteins encoded in 1385 genomes containing 2575 replicons (1483 chromosomes and 1092 plasmids) with these profiles using Hmmer 3 [86]. Replicons either had hits to the majority of the protein profiles (Figure 1B), or to only few of them. In the latter case, the profiles retrieved homologs of the secretin and of the ATPase in other cell machineries. We filtered these hits, retaining hits that were statistically significant. We then selected hits from replicons containing a minimal number of the 12 gene families (different criteria to infer NF-T3SSs or flagella, Materials and Methods), and those hits had to co-localize in units of two different core genes or more (Materials and Methods). Subsequent expert analysis, informed by the available literature, resulted in a dataset of 921 putative T3SSs: 222 NF-T3SSs and 699 flagella distributed among 155 and 642 genomes, respectively. In the following sections, we summarize 216 NF-T3SSs, after excluding six on the basis of phylogenetic analyses (Table S2). We also analyzed a subset of 357 flagellar T3SSs that were representative of the diversity in the entire dataset and which had been manually curated (Materials and Methods, Table S3, Dataset S2). The vast majority (92%) of NF-T3SSs were encoded in chromosomes. NF-T3SSs were only identified in bacteria with outer and cytoplasmic membrane (diderms): Chlamydiae, Proteobacteria, and Verrucomicrobia. Flagella were found in many more taxa, within diderms (Acidobacteria, Aquificae, Bacteroidetes, Chloroflexi, Deferribacteres, Gemmatimonadetes, Nitrospirae, Planctomycetes, Proteobacteria, Spirochetes, Thermotogae and Verrucomicrobia), and also among bacteria lacking an outer membrane (monoderms: Actinobacteria and Firmicutes). These results confirm previous studies showing that the possession of NF-T3SSs by bacterial taxa is much more restricted than flagella [79].

Some of the T3SS loci lacked core genes, but functional T3SS genes need not be in a single locus. We therefore investigated in detail the replicons with partial T3SS loci. 61 replicons had at least three of the nine NF-T3SS core genes homologs but lacked at least two of them. Only two of these systems corresponded to NF-T3SS and all others were of flagellar origin (Figure 1B). This finding suggests that NF-T3SSs are rapidly deleted when they lose function. Incomplete loci might represent NF-T3SSs encoded in multiple loci, systems with different functions or systems undergoing genetic degradation. For example, a cryptic short NF-T3SS locus in *E. coli* (ETT2 locus) [87] often lacks *sctT* and genes encoding structural proteins such as *sctL* and *sctD*. ETT2 was suggested to play an important role in virulence by regulating the NF-T3SS of the locus for enterocyte effacement (often termed LEE) [88], [89]. We then investigated the replicons lacking at most two of the nine core genes.

Of 216 such NF-T3SSs, 21 included complete NF-T3SSs scattered in the genome and 10 corresponded to a Myxococcales system (“Myxo”) described below. As previously reported, the NF-T3SSs are spread over at least four loci in *Chlamydia*, three of which contain core genes [81], [90]. Scattered NF-T3SS loci are present in all Chlamydiales genomes (“Chlamy” systems), in *Hamiltonella defensa* [91], an intracellular Proteobacterium, and in the plasmid pYPTS01 of *Yersinia pseudotuberculosis* PB1/+. We note that the NF-T3SS is constitutively expressed in all development stages in *Chlamydia* [92]. Absence of specific regulatory elements might alleviate selection for the clustering of all NF-T3SSs genes and facilitate the fixation of rearrangements scattering the NF-T3SS into several loci. These disrupted loci are less likely to be transferred horizontally because acquisition of a complete system would require the co-transfer of different regions of a replicon.

**Figure 2:**
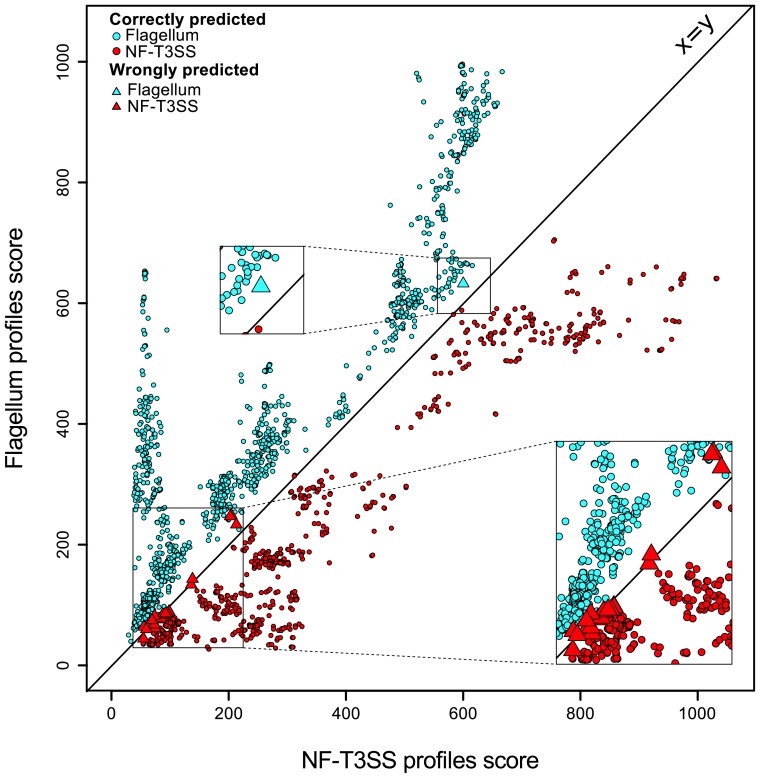
Discrimination between NF-T3SSs and flagella based on Hmmer profile scores. Each T3SS core protein was scored using the protein profiles for flagella (Y axis) and for NF-T3SSs (X axis). One sees a clear separation around the main diagonal. The color indicates the system (blue for flagellar proteins, red for NF-T3SS proteins). A circle indicates a correct prediction, and a triangle a wrong prediction. The analysis is described in Materials and Methods, and the learning dataset is shown in Figure S1.

We tested the quality of the discrimination between NF-T3SSs and flagella by protein profiles with linear discriminant analysis (Figure 2, Figure S1). Our results indicate that the accuracy of assignment was >97% in gene-by-gene analyses, and >99% for combined protein profiles (see Materials and Methods), and that all mis-classified NF-T3SSs were in Myxo and Chlamy systems (see below). The accuracy of discrimination between the two types of T3SS for single proteins shows that these profiles are potentially useful for unassembled genomic data, including metagenomic data. We have therefore implemented a web server that allows detection of NF-T3SS and flagellar genes with our profiles (http://mobyle.pasteur.fr/cgi-bin/portal.py#forms::T3SSscan-FLAGscan). The NF-T3SSs described here can be queried and visualized from http://secreton.web.pasteur.fr.

### The origin(s) of T3SS

**Figure 3:**
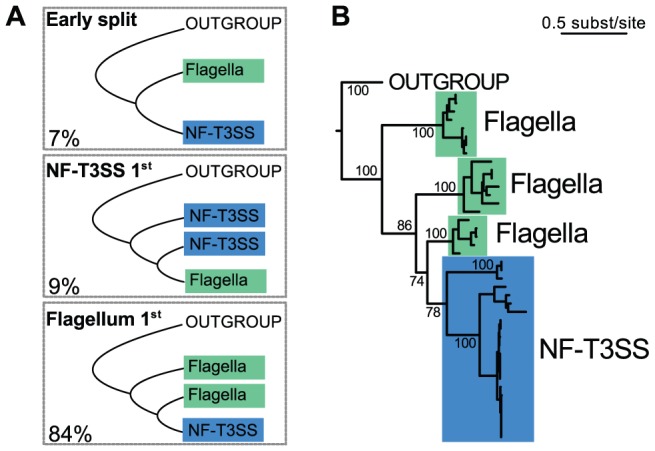
Rooting the history of T3SS. (A) Representation of the three possible scenarios for the relative emergence of the flagellum and of the NF-T3SS. The numbers represent the percentage of each scenario in the bootstrap analysis of the ATPase family (out of 997 bootstrap trees where outgroup sequences were monophyletic, Figure S2, Protocol S1, Text S1). (B) The maximum likelihood tree of the ATPase family shows that the NF-T3SS derives from the flagellum. Support values are shown only for relationships important to discriminate between the three scenarios in the panel (A). They correspond to the occurrence of the splits in 1000 bootstrap replicates. The multiple alignment used to build this tree had 296 informative sites and is supplied in Dataset S1.

We now turn to the evolutionary origins of the T3SS, a topic that has been extensively debated [75], [82], [93], [94], in order to decide between three scenarios: the early split of the two systems, the flagellum-first or the NF-T3SS-first hypotheses (Figure 3A). Our analysis showed that of the T3SS proteins with clear homologs between flagella and NF-T3SSs, only the ATPase also has homologs in other cell machineries with significant sequence similarity to allow rooting the T3SS tree. A set of homologs of the T3SS ATPase and F- and V- ATPase catalytic subunits [39] (Protocol S1) were selected and aligned, resulting in sequences with an average length of 459 amino-acids. We selected 296 informative positions from the multiple alignment with BMGE [95] (Dataset S1), and chose the tree with the highest maximum likelihood from 200 phylogenies built with RAxML [96]. This tree supports the T3SS monophyly with high support (100% bootstrap), and places the root of the T3SS tree within flagellar sequences (Figure 3B, Protocol S1). NF-T3SS sequences emerge in one clade within the flagellar T3SSs. We also counted the proportion of the 1000 bootstrap trees fitting each of the three evolutionary hypotheses (Figure 3A, Protocol S1). 84% of the trees support the flagellum-first hypothesis, arguing strongly against an early split between flagella and NF-T3SSs or that flagella were derived from an NF-T3SS. We confirmed this result by an analysis on a larger dataset including all curated systems. It is often difficult to obtain clear bootstrap supports for inner branches in very large trees spanning sequences with limited similarity and/or few sites. However, even this very large dataset clearly supported the flagellum-first scenario (>72% of the bootstrap trees, Text S1, Figure S2).

### The phylogeny of NF-T3SS

**Figure 4:**
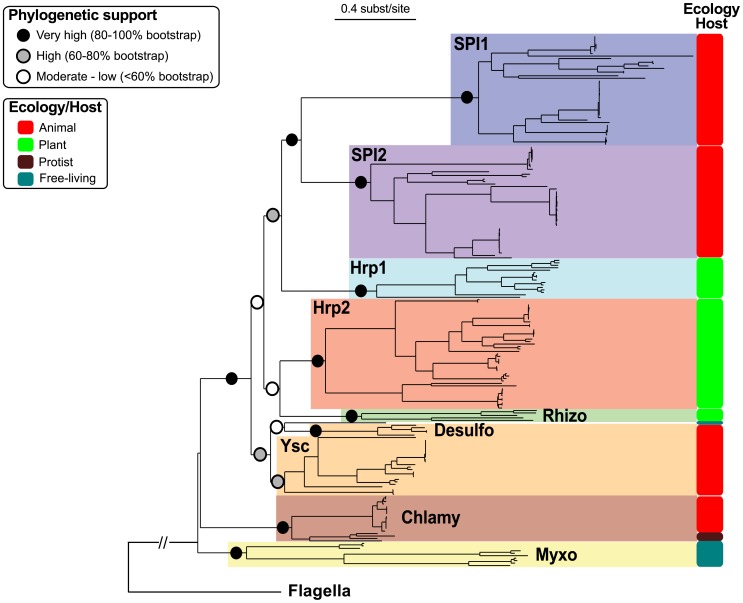
Concatenate phylogeny of the core NF-T3SS proteins with flagellar homologs. The eight individual gene trees are displayed in Figure S3. The large monophyletic clades highlighted by different colors correspond to the NF-T3SS families (names in bold, see Text S2). Colored circles indicate the bootstrap support of these families and of the relationships between them. The “Ecology/Host” panel indicates the predominant bacterium/eukaryote associations for each family (see main text and Figure S4). The external branch indicates the position of the flagellum sequences and was inferred with the “rooted” dataset (“rooted” tree is in Figure S5). “Chlamy” stands for Chlamydiales, “Desulfo” for Desulfovibrionales, “Myxo” for Myxococcales, and “Rhizo” for Rhizobiales. The scale of the tree is given in substitutions per site.

In order to investigate early steps in the evolution of the NF-T3SS we reconstructed the phylogenies of the eight core proteins present in both NF-T3SSs and flagella for both single genes and their concatenated sequences. Initially we included the flagellar proteins to root the NF-T3SS tree. Then we restricted the analyses to the NF-T3SS proteins to obtain longer and more conserved multiple alignments allowing more accurate phylogenetic inference. The individual phylogenies of the eight core proteins support a common descent of all NF-T3SSs from a single ancestor and identify the same NF-T3SS groupings (Figure S3, Table S4). These groupings extend and clarify a previous classification of NF-T3SSs [62] (Figure 4, Figure S4, Text S2). The use of the program Prunier [97] showed that any topological differences among the eight individual gene trees and the concatenate trees were supported by less than 90% of the bootstraps (Text S3). Thus, gene-wise and concatenated “rooted” and “unrooted” phylogenetic analyses all support a similar history for the eight core NF-T3SS genes showing that they evolved together, apart from their flagellar homologs.

### The first NF-T3SS was probably not a contact-dependent secretion system

**Figure 5:**
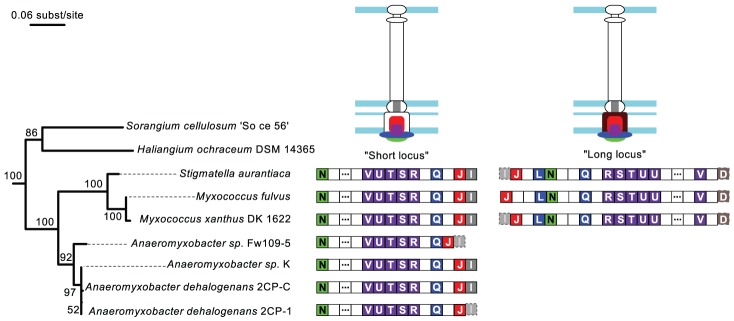
Genomic organization of the Myxo NF-T3SS. The gene contents of the “short” and “long” Myxo NF-T3SSs are displayed on the 16S rRNA phylogeny of the group. The 16S rRNA maximum likelihood tree was built with RAxML [96] (General time-reversible model + 4-categories-discretized Gamma distribution for rate variations among sites) and chosen from the best of 200 starting trees. The numbers in the tree nodes refer to bootstrap values out of 100 replicates. The identified NF-T3SS genes are represented by boxes containing the last letter of the “*sct*” gene name, and colored according to the color code of Figure 1A. Dashed transparent boxes correspond to genes showing hits for Hmmer sequence profiles with i-evalue above the 10^−3^ threshold. Empty boxes correspond to genes not annotated in our analysis, and boxes with three dots correspond to several (4 to 16) consecutive non-annotated genes. Proteins encoded in the loci are colored on the NF-T3SS structure drawings, whereas parts of the structure whose corresponding gene could not be found remain unfilled.

The NF-T3SS tree places the root between one group of systems found in Delta-proteobacteria of the Myxococcales order (the “Myxo” group), and all remaining systems (≥98% bootstrap support, Figure 4 and Figures S4, S5). The early diverging Myxo group includes both a long and a short variant of the NF-T3SS locus. These variants lack core NF-T3SS genes, resulting in their prior annotations within the genome of *Myxococcus xanthus* as relics of NF-T3SS undergoing degradation [98]. If this interpretation were correct, then these genes should evolve quickly and our assignment of these variants to a basal phylogenetic position might be artifactual. However, we identified six additional genomes from the same clade (Cystobacterineae) having homologous systems (Figure 5) (see [99] for the taxonomy of Myxococcales). All fully sequenced genomes of Cystobacterineae have the “short” locus and a monophyletic group of three of these genomes also possesses the “long” locus. These loci have G+C contents within the 25–75% range of the G+C genomic content (SeqinR [100] analysis with a 1 kb sliding window), and a conserved gene order (Figure 5). The core genes that are common to these variants correspond to the secretion apparatus, the ATPase, the smaller inner ring protein and the inner rod protein (short locus). The long locus also contains the large inner ring protein. These core proteins interact with each other and also correspond to the most conserved NF-T3SS core proteins. The long and short loci each correspond to distinct monophyletic groupings (Figure S4) that have probably been inherited vertically because the topology and branching structures of these phylogenies resemble those of the 16S rDNA tree (Figure 5). The conservation of these systems in sequence, gene composition and genetic organization is striking because the species harboring them diverged a long time ago. For example, the 16S rDNA subunits of *Anaeromyxobacter* and *Myxococcus* show lower sequence similarity than that between *Escherichia coli* and *Vibrio cholerae*. The strong conservation of these loci in sequence and organization over such long time scales suggests that they are functional.

We argue that the Myxo NF-T3SSs probably derived from ancestors that were neither flagella nor protein delivery systems. Myxo systems lack proteins that are indispensable for flagellar function, such as FlgBCDEK, FliEG, FlgH and MotAB. They also lack NF-T3SS genes such as the secretin, the major needle subunit (SctF), the tip protein (LcrV) and the translocon (YopB/YopD). Homologs of the tip and translocon proteins may have been missed in our sequence similarity searches because of their rapid evolution. However, the flagellar proteins, the secretin and SctF are highly conserved and should have been found by our sequence similarity searches had they been present. Our profiles show significant sequence similarity between SctF, the major needle subunit of the NF-T3SS, and FliC/FlgL (the flagellin and a hook-associated protein), whose homology was previously suggested based on structural data [71], [101]. Hence, the earliest NF-T3SS probably contained an ancestral SctF that was lost in Myxo systems. The lack of an outer membrane channel and of SctF suggests that Myxo systems are not able to deliver effectors to eukaryotic cells and possibly not even to secrete proteins to the extracellular space.

### Multiple origins of secretins

**Figure 6:**
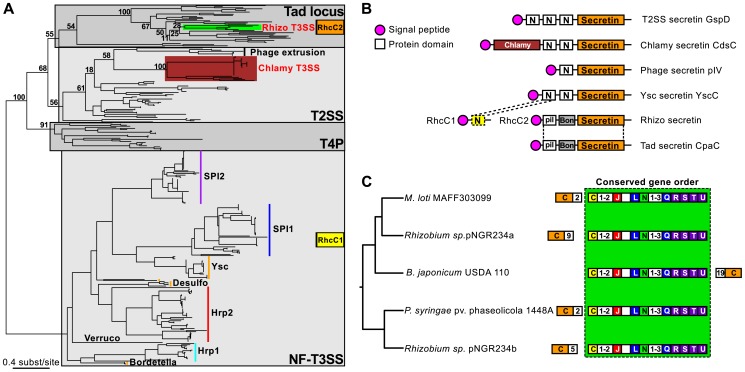
Evolutionary history, domain architecture, and genetic organization of the NF-T3SS secretins. (A) Phylogeny of the secretin family. The numbers represent bootstrap support values for branches separating the different types of secretins found in NF-T3SSs (highlighted by colored bars). Gray boxes indicate the main secretin groups. The two genes encoding the Rhizo secretin are indicated in orange and yellow boxes (see panel B). NF-T3SS types are named as in Figure 4. (B) Domain architecture of the secretins related to NF-T3SS secretins. PFAM domains were identified using InterProScan [140], except for the Chlamy-specific N-terminal domain of the NF-T3SS secretin CdsC (burgundy box), and the dashed box “N-domain”, which were identified by sequence similarity with the other secretins using Blast. Signal peptides were detected with InterProScan and PsortB [141]. Dotted lines correspond to the boundaries of the alignments of Blast hits. See Text S4 for more analyses of Rhizo secretins. (C) Analysis of the genetic organization of the Rhizo loci mapped on their phylogeny. The phylogeny was inferred from the concatenation of the eight NF-T3SS core genes (Figure 4, Figure S4). Conventions are the same as in Figure 5. Numbers in white boxes correspond to the number of genes separating the block of genes with conserved order (light green box) from the RhcC2 secretin (outside the box), or to the number of consecutive genes not annotated in this study (inside the box).

We next analyzed the diversification of NF-T3SS within the main branch of its phylogeny, i.e. among loci including a secretin. This branch includes all NF-T3SSs shown experimentally to deliver effectors into the eukaryotic cytosol. The first split along this branch separates the Chlamy from the other systems with 100% bootstrap support (Figure 4, Figure S3, Table S4). Subsequent diversification was very rapid within the other taxa as shown by a succession of short branches with weak bootstrap support (Figure 4 and Figures S3, S4, S5). To investigate the early NF-T3SS diversification we made a phylogenetic analysis of the NF-T3SS secretin together with secretins from T2SS, T4P, Tad system and filamentous phages [102]. Surprisingly, the phylogeny shows that secretins have been independently recruited to the NF-T3SS on at least three occasions (Figure 6A). The Rhizo secretin RhcC2 branches together with secretins from the Tad loci (e.g. RcpA and CpaC) [103]. The secretin domain of Chlamy NF-T3SSs (excluding a large unique N-ter region, see Figure 6B and [81], [92]) clusters with the *gene IV* secretin of filamentous phages within T2SS secretins. The secretins from the remaining NF-T3SSs cluster together in a third group (Figure 6A) hereafter referred as “NF-T3SS-like” secretins. The most parsimonious explanation for these results is that the last common ancestor of extant NF-T3SSs lacked a secretin.

The secretin of Rhizo NF-T3SSs is encoded by two genes, *rhcC1* and *rhcC2* (Figure 6B) [104], and we show that they have distinct origins. The gene *rhcC2* encodes a protein whose architecture is similar to the secretin of the Tad locus of *Caulobacter crescentus* CpaC (Figure 6B, Text S4), in agreement with its phylogenetic position within Tad loci (Figure 6A). It includes the “secretin” domain, absent from RhcC1, and an N-terminal “BON” domain [105]. The gene *rhcC1* is found at a conserved position in Rhizo loci, among other NF-T3SS core genes (Figure 6C). The protein RhcC1 is homologous to the N-terminal part of the NF-T3SS-like secretins (Blast search with e-value<10^−3^, Figure 6B, Text S4), which includes the first “N-domain” (PFAM PF03958) of these secretins (Figure 6B). This demonstrates that RhcC1 has a common origin with NF-T3SS-like secretins. Therefore, both protein domains analyses and phylogenetic analyses agree in suggesting the independent acquisition of the secretin in the ancestral Chlamy, and in the ancestor of all the other NF-T3SSs. According to this interpretation, the ancestral Rhizo system had originally an “NF-T3SS-like” secretin, RhcC1, and secondarily acquired the secretin RhcC2 from a Tad locus. The recruitment of this second secretin was accompanied by the deletion of a large C-terminal fraction of the original secretin, and is thus an example of a partial homologous gene replacement.

### Diversification of NF-T3SS and host interactions

The association between the repertoire of NF-T3SS genes and the nature of the eukaryotic host maps well on the phylogenetic tree of the NF-T3SS (Figure 4, see Figure S4 and Text S2 for more details). Notably, there is a clear distinction between systems involved in interactions with animals or protozoa, on one hand, and plants and fungi on the other (Figure 4 and Figure S4). This is not necessarily expected because the phylogeny was based on ubiquitous, highly conserved, core genes that are not expected to drive the ecological diversification of the NF-T3SS. Indeed, *Chlamydia* effectors can be recognized and exported by the very distant NF-T3SS of *Shigella* [106], and effectors of animal-associated NF-T3SS can be exported by plant-associated NF-T3SS [107]. Therefore, the specificity of the ecological interaction between the NF-T3SS and the eukaryotic cell is caused by the diversification of extracellular components such as the needle and the tip [26] (see the gene content panel on Figure S4). Hence, the clear separation of NF-T3SSs in terms of host cells in the phylogenetic tree of core genes likely reflects their genetic linkage to genes encoding the extracellular proteins of the NF-T3SS.

The classification of the NF-T3SSs may help unravel unknown ecological interactions between free-living bacteria and eukaryotes (Figure 4, Figure S4). *Shewanella* spp. are free-living marine bacteria. They are not pathogenic for fish, but are a major cause of marine seafood spoilage [108]. Their NF-T3SS system, SPI2, is closely related to that found in *Edwardsiella* spp. which are true fish pathogens. We did not find any reports of specific interactions between *M. mediterannea* and eukaryotes, but this bacterium belongs to the microbiota of *Posidonia oceania* [109], a marine angiosperm, and encodes a plant-related NF-T3SS (Hrp1). In some cases the associations between the NF-T3SS type and the host seem less clear-cut. *X. albilineans*, unlike other *Xanthomonas* phytopathogens, has one SPI1 system, which does not seem to be involved in pathogenesis, even though this bacterium proliferates in the xylem of plants [110]. *Salmonella enterica* encodes animal-related NF-T3SSs that are necessary for the infection of both animals and plants by this bacterium [111]. *Hahella chejuensis*, a marine Gamma-proteobacterium, is a potential biological agent to fight algae [112] but it is also part of the goat milk microbiota [113], and encodes two Ysc NF-T3SSs of unknown function. Interestingly, such mismatches between NF-T3SS and eukaryotic host (Figure S4) are only present in animal-protozoa systems that are able to interact with plants. Their study might provide clues on the historical adaptation of the ancestral NF-T3SS, which encoded a needle, to a cell-wall adapted pilus that can interact with plants. These observations also suggest a certain degree of flexibility in the interactions of NF-T3SSs with plant and animal hosts.

## Discussion

The phylogeny of the ATPase, the multiple recruitments of secretins and the finding of highly conserved basal NF-T3SSs qualitatively change our views on the evolution of the NF-T3SS. In this work we provide independent lines of evidence showing that the flagellum was primordial and that the NF-T3SS arose *via* exaptation. Firstly, the ATPase tree robustly roots the T3SS tree within flagella (Figure 3). Secondly, confirming prior results [93], the phylogenetic distribution of the flagellum is much broader than that of the NF-T3SS. Thirdly, our data suggests that the last common ancestor of the NF-T3SSs lacked a secretin. Hence, effector delivery into eukaryotic cells was probably not an ancestral trait of NF-T3SS.

### T3SS exaptation

**Figure 7:**
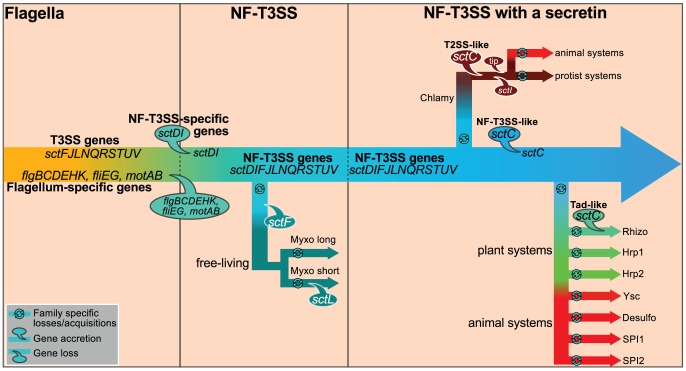
Proposed scenario for the evolution of NF-T3SS. We propose that genes common to the flagellum and the NF-T3SS were present in the flagellar ancestor of NF-T3SS. They are designated as “T3SS genes”. Then we detail the evolution of this system to the extant NF-T3SSs. First, the loss of flagellum-specific genes resulted in loss of the motility function. Presumably this system kept the ability to translocate or secrete proteins. The ancestral NF-T3SS experienced a series of gene losses and gains while diversifying to the ancestor of all extant NF-T3SSs. One early lineage derived into the Myxo systems by loss of some genes, notably SctF. Acquisition of secretins and of a few other genes allowed the formation of ancestral contact-dependent protein secretion systems, and the concomitant ability to subvert eukaryotic cells by direct delivery of effectors in their cytosol. Secretins were recruited to the NF-T3SS at least three times from three different cell machineries. Finally, the NF-T3SS quickly diversified and adapted to different host cells. Some components of NF-T3SS, such as the translocon proteins (YopB/YopD), the needle length determinant (SctP) or the needle tip (LcrV), cannot yet be integrated in this schema because of low or undetectable sequence similarity among T3SSs. Structural and sequence similarities were previously noted between translocon proteins of *Chlamydia* (CopB) and *Salmonella* (SipB) [142]. In contrast, we found no more than 28% identity over less than half of the protein when aligning CopB with all complete genomes in GenBank after excluding Chlamy proteins (Blastp, e-value>0.05). We also did not find significant sequence homologs of YopB/YopD in the NF-T3SS of Myxo or plant-associated bacteria. Since the translocon is required for protein delivery but not for secretion [35], it might have been acquired after the secretin. Balloons indicate gene losses and accretions. Only genes mentioned in the main text are shown. Abbreviated names of taxa are as in Figure 4.

Our insights also allow us to propose a scenario for the evolution of the NF-T3SS (Figure 7). Initially, T3SS evolved to transport extracellular flagellar components [56]. The flagellar T3SS is not only an essential component of the flagellum but variants have also evolved to transport other proteins, e.g. virulence factors in *Campylobacter jejuni* and *Y. enterocolitica* [114], [115]. In the endomutualist non-motile *Buchnera* spp., the locus encoding flagellum components is reduced to the basal body, which has been proposed to serve as a protein secretion system [116], [117]. Interestingly, flagella-like loci have been detected in the genomes of a dozen species that are not thought to have flagella [74]. These observations suggest that flagellar T3SSs have been exapted on multiple occasions for secretion of proteins unrelated to the flagellum. However, NF-T3SSs are monophyletic, indicating that their exaptation from the flagellum only occurred once. During experimental evolution, mutations that induce a loss of function are among the most frequent adaptive events detected [118]. We would then anticipate that exaptation to the NF-T3SS would have been accompanied by deletions of flagellum-specific genes. Yet the core function of the T3SS as a facilitator of protein translocation was probably never lost: (i) the secretion apparatus is highly conserved; (ii) the needle protein, which is translocated by the T3SS, is homologous to flagellar proteins; (iii) experimental evidence shows that extant NF-T3SS effectors can be secreted by the flagellum T3SS [119].

Our results contradict the proposal that NF-T3SS evolved in Chlamydiales before being transferred to Proteobacteria [62], [93]. Transfer of the multiple loci within the Chlamy system would require multiple events, whereas the unique loci of other NF-T3SSs can be transferred in one single event. All Chlamy NF-T3SSs include a T2SS-like secretin whereas other NF-T3SSs share a secretin from a distinct origin (Figure 6). Finally, key elements between these systems have no homologs, e.g. the proposed tip protein for Chlamy (CT584) cannot be aligned to tip proteins from other systems [120]. Hence, we propose that the last common ancestor of the NF-T3SSs derived from a flagellum, lacked a secretin and included a periplasmic or extracellular structure based on SctF. Interestingly, Spirochetes have periplasmic flagella, and in some cases lack the outer membrane protein FlgH [121]. Inactivation of the secretin in *S. enterica* SPI1 NF-T3SS prevents the formation of extracellular appendages but does not prevent the secretion of the main needle filament subunit (SctF) to the periplasm, where it remains associated with the inner ring proteins [53]. Hence, it is conceivable that a needle-like structure might have assembled at the periplasm in the ancestral NF-T3SS even in the absence of an outer membrane channel. We argue that this system was subsequently transferred across taxa prior to independent acquisitions of a secretin.

### The atypical Myxo system

The early evolution of the NF-T3SS was accompanied by the accretion of new genes that are present in even the most basal clades of the NF-T3SS (Figure 7). These include the structural genes *sctD* and *sctI* and probably also system-specific chaperones and regulators, whose evolutionary patterns are obscured by their fast evolutionary rates. The Myxo NF-T3SS was the first group to split from the others (Figure 7). Myxo systems are only present in Cystobacterineae and should have significant adaptive value because they are present in all available genomes of this taxon, show strict vertical inheritance and are highly conserved in sequence and genetic organization. The absence of the secretin and translocon as well as other proteins essential for protein delivery by other NF-T3SSs suggests that the Myxo NF-T3SS is unable to deliver effectors into the cytosol of eukaryotes. This is consistent with Cystobacterineae ecology, which are free-living bacteria that prey on other bacteria [122]. These systems could thus be involved in protein translocation and/or make some sort of periplasmic or extracellular structure. Functional analysis of these systems would be needed to understand their function.

### Multiple recruitments of secretins to NF-T3SSs

Acquisition of the secretin was the next major step in NF-T3SS evolution for which clear evidence is available. Our analysis shows that the secretin was recruited three times: in Chlamy (from T2SS), in the other systems (possibly from T4P) and then again in Rhizo (from Tad systems). This is consistent with the remarkable versatility of secretins as outer-membrane channels [123]. The interaction between the inner membrane rings and the secretin takes place between the C-terminal region of SctD and the N-terminus domains of the secretin [124]–[127]. Interestingly, the only part of the early secretin RhcC1 that remains in Rhizo after its partial replacement is the N-terminal domain that interacts with SctD (Figure 6, Text S4). The Tad and the RhcC2 secretins lack this N-terminal domain and have a membrane-interaction, “BON” domain [105], that is present in SctD but absent in other secretins. This domain architecture might interfere with a direct interaction between RhcC2 and SctD, which led to the conservation of RhcC1 as a linker between the two. In that case, interactions of the secretin during the assembly of the NF-T3SS might be critical for successful acquisitions of secretins from other systems. Flagellum assembly resembles the inside-out model of NF-T3SS assembly [128], [129]. The ancestral NF-T3SS probably also assembled inside-out because it lacked a secretin. The inside-out assembly of the secretion apparatus and especially of the newly acquired SctD protein at the inner membrane might have led to interactions with secretins from other systems that stabilized an outer membrane channel in the NF-T3SS complex. Indeed, extant NF-T3SS secretins are inserted in membranes independently of the assembly of the inner ring. The inner ring assembles, and then stabilizes the secretin multimer [53]. These interactions presumably evolved towards a stable genetic linkage of the secretin with the NF-T3SS. Outside-in assembly modes, e.g. as proposed for *Yersinia* NF-T3SS, might have evolved after the acquisition of the secretin. Studies on the assembly of Myxo systems might therefore elucidate the mechanisms of assembly of the ancestral NF-T3SS and also of the evolution of the assembly process of the other NF-T3SSs.

### Rapid adaptation of NF-T3SSs to diverse eukaryotic cell envelopes

Acquisition of the secretin and of the translocon proteins allowed bacteria to deliver effectors to eukaryotic cells. This was followed by very rapid diversification of NF-T3SSs into groups with different characteristics and tropisms (Figure 7). Such rapid radiation hinders robust phylogenetic inference after the split of the Chlamy systems at the base of the remaining NF-T3SSs (Figure 4), even though we were able to infer older splits with strong confidence. Needle/pilus, tip and translocon proteins, all of which promote intimate interactions with the host, evolved quickly and were probably key determinants of NF-T3SSs radiation. The original NF-T3SS included a needle (SctF) resembling the flagellum homolog. The replacement of the needle by a pilus was a key late adaptation in NF-T3SSs that specifically interact with the cell walls of plants and fungi. Sequence evolution of the NF-T3SS pilus of some plant-associated bacteria is driven by strong positive/diversifying selection in response to host defenses [130], [131]. It might be expected that vertebrate immune systems would have had similar effects on the evolution of animal-associated NF-T3SSs. Hence, diversifying selection on plant-associated NF-T3SSs is probably not the cause of the differences between pili and needles. Instead, the pilus probably reflects adaptation to the thick cell walls of plants and fungi. More genomic, functional and structural data on NF-T3SSs involved in plant interactions will be necessary to understand whether all these plant-associated NF-T3SSs arose independently or derived from the same ancestral NF-T3SS.

This work shows how a key protein secretion system arose from the recruitment of a structure that evolved for another purpose. The T3SS was initially adapted to the transport of flagellar components through the membrane and was probably exapted on multiple occasions to transport other proteins. One of those exaptations resulted in the ancestral NF-T3SS. This system adapted to its new function by a series of gene losses and acquisitions. Strikingly, it acquired secretins multiple times. Thereafter, the system was able to deliver effectors directly to eukaryotic cells, which dramatically increased its rapid diversification due to the adaptive value of NF-T3SS for mutualistic and antagonistic interactions with eukaryotes. This evolutionary reconstruction represents a remarkable example of how successive losses and recruitments of components from a series of existing molecular machines can lead to the evolution of a new complex system.

## Materials and Methods

### Genome data

Genomes were extracted from GenBank Refseq. To extend the taxon sampling, we added to the dataset some draft genomes of interest. We analyzed 1385 genomes with 2575 replicons (1483 chromosomes and 1092 plasmids).

### Data availability

The NF-T3SS clusters identified in this study can be queried using different criteria (including taxonomy and NF-T3SS family name), and visualized along with the results of our Hmmer profiles searches at http://secreton.web.pasteur.fr. The profiles of NF-T3SS and flagellum proteins can be queried at http://mobyle.pasteur.fr/cgi-bin/portal.py#forms::T3SSscan-FLAGscan. The list of NF-T3SSs and flagella included in phylogenetic analyses can be found in Dataset S2.

### Construction of sequence profiles for NF-T3SSs and flagella

We selected one sequenced model organism from each described NF-T3SS family (genomes marked in red on Figure S4, list in Table S5 [6]). We extracted NF-T3SS protein sequences according to their genome sequence annotations and the literature. We performed similarity searches between these sequences with a Blast “all against all” search and applied a clustering algorithm with stringent parameters on the transformed e-value (-log(e-value), MCL [132] inflation parameter I = 1.5) to sequences showing hits with an e-value lower than 10^−3^. We obtained nine families that were found in all model systems, which corresponded to the nine previously described NF-T3SS core proteins: SctC, SctJ, SctN, SctQ, SctR, SctS, SctT, SctU, SctV. We aligned these nine protein families with Muscle [133], manually edited the alignments with Seaview [134], and built sequence profiles with Hmmer [86]. A similar approach was conducted for flagella from phylogenetically distinct model organisms (MCL clustering, I = 1.8) (List in Table S3). Out of 14 protein families widely conserved in flagella, eight were homologous to NF-T3SS core proteins (Table S1, protein clustering of protein families obtained from NF-T3SS and flagellar model systems, MCL parameter I = 2.5), and were extracted to build Hmmer sequence profiles. We also selected three widely conserved flagellar families with no NF-T3SS homolog (confirmed by the clustering above and Hmmer profile searches): FliE, FlgB, FlgC (rod proteins), and built sequence profiles to identify other occurrences of these proteins. Additional profiles were also built for FlgDEKL, FliG, MotA and MotB (MCL parameter I = 1.5) that are essential flagellar-specific genes [71].

### NF-T3SSs/flagella identification

To discriminate between homologous genes in flagella and NF-T3SSs, we performed a Hmmer search with the profiles of the eight proteins shared between them (“core proteins”), the secretin (the NF-T3SS-specific core proteins), and the three selected flagellum-specific proteins. Hmmer hits with Evalue and best-1-domain Evalue (or i-evalue) lower than 10^−3^ were selected. Two hits were said to be contiguous when separated by less than 35 genes (average size of a flagellum cluster). We searched for clusters of contiguous hits to separate NF-T3SSs and flagellar systems. Clusters of genes showing positive hits for at least seven of the eight core genes, including a secretin, and lacking all three flagellum-specific genes were classed as NF-T3SSs. Sets of clusters of NF-T3SS core genes (core genes+*sctC*) not containing flagellum-specific genes were inferred as scattered NF-T3SSs. Flagellum clusters contained no secretin and had hits for at least 10 flagellar genes (core genes+flagellum-specific genes). Scattered flagella had hits for at least 10 flagellar genes, and had at least one cluster containing flagellum-specific genes with one core gene. All detected NF-T3SSs, plus other clusters close to the definition above (i.e. having fewer of the core genes clustered) were manually curated and checked according to the available literature. Thus, the Myxo systems were retrieved even if they lacked a secretin and flagellum-specific genes. We did not include in our analysis the system of *Lawsonia intracellularis* because its flagellar genes and NF-T3SS genes were intermingled in different positions of its genome, rendering the reconstruction of the two systems more hazardous (Table S2).

### Discriminant analysis with hits for both NF-T3SS/flagellar genes

We attributed two types of Hmmer scores to genes with homologs in both systems: one score corresponding to the NF-T3SS profile and the other to the flagellar profile. We made a learning dataset including a third of the T3SS genes and their predicted role (NF-T3SS or flagellum). This subset was randomly drawn from the set of all T3SS. Using linear discriminant analysis [135], we predicted the type of system for the remaining two thirds of the dataset. We performed such an analysis with the combined dataset (all eight genes common to the flagellum and NF-T3SS together) as described above, and also in a gene-by-gene analysis. Accuracy is defined as the number of true predictions over the total number of predictions.

### Individual phylogenetic analyses

We extracted from the genomes the genes encoding proteins homologous to T3SS core genes that were detected as part of a NF-T3SS or flagellum system. In a given system, when multiple Hmmer hits were available for a single gene, we kept the one displaying the lower Evalue and the maximal length. Many flagellar systems had multiple hits for the same genes scattered in the genome. We manually curated a subset of these flagella (357 out of 699 detected, the list of strains is in Dataset S2). We aligned sequences with Muscle (default parameters, [133]) and selected informative sites with BMGE (BLOSUM30 similarity matrix, gap rate cut-off = 0.20, sliding window size = 3, entropy score cut-off = 0.5 [95]). We built phylogenetic trees with RAxML ([96], Le and Gascuel [136] matrix + 4-categories-discretized Gamma distribution for rate variation among sites + empirical frequencies of amino-acids): we selected the best maximum likelihood tree among 200 different starting tree inferences, and computed 1000 bootstrap trees (i.e. trees based on bootstrap alignments, consisting of randomized sites drawn with replacement from the original alignment, and of the same size of the original alignment). In the case of the ATPase SctN, we built an extra dataset that we extended with previously described outgroup sequences [39] (see Text S1) and built a tree as described above. We also ran an extra phylogenetic analysis in a similar way on a subset of these sequences (see Protocol S1, Text S1). We built a tree as indicated above with a secretin dataset that included i) sequences identified in a previously described dataset [102] that were retrieved using their accession numbers; ii) SctC of detected NF-T3SSs; iii) all the secretins we found in Myxo and Chlamy genomes. Sequences displaying branch lengths longer than 1 substitution per site were excluded from phylogenetic analyses, and the phylogenetic reconstruction was run again with the cleansed dataset. This led to the exclusion of several flagellar systems and of five potential NF-T3SSs. Some of these systems are probably undergoing degradation (Table S2).

### Concatenated phylogenetic analyses

The protein alignments of the genes were concatenated and phylogenetic trees were built with RAxML [96] (Le and Gascuel matrix [136] + 4-categories-discretized Gamma distribution for rate variation among sites + empirical frequencies of amino-acids) with 100 rapid bootstraps [96] for the rooted tree. We made a more thorough phylogenetic search for the unrooted dataset: we performed 100 bootstrap replicates, and mapped them on the best (i.e. with the highest likelihood) among 100 phylogenies obtained from distinct start trees. We attributed families to predicted NF-T3SSs according to previously defined families ([62]; list of genomes in [6]). We searched *a posteriori* for putative significant inconsistencies between phylogenies of individual unrooted trees and the concatenate tree using the program Prunier [97] (bootstrap threshold = 80% and 90% in both gene trees and in the reference tree, see Text S3).

### Building families of neighboring genes

We extended the cluster of NF-T3SS core genes by 10 genes upstream and downstream in the replicon sequence. All these protein sequences were extracted, and a similarity search (Blast “all against all”) was performed between them. Pairs of sequences having hits with e-value lower than 10^−3^ were clustered based on Blast alignments using the Silix program ([137], parameters used: minimal percentage identity = 20, minimal percentage of sequence overlap = 50, and minimal accepted length for sequences = 50). The most abundant protein families, considering both replicons and NF-T3SS families, were extracted and Hmmer profiles were built from them to extend the search in NF-T3SS neighboring genes.

### Programs used for graphics

We used the Scriptree program [138] to draw annotated trees (Figure 4; Figures S3, S4, S5) and Figtree (http://tree.bio.ed.ac.uk/software/figtree) to draw trees (Figure 3B, Figure 5, Figure 6). Graphics on Figure 1B, Figure 2, and Figure S1 were drawn with R (http://www.r-project.org). All figures were modified with Inkscape (http://www.inkscape.org).

## Supporting Information

Dataset S1 The filtered alignment of the ATPase family used for phylogenetic analyses and the tree in Figure 3B (Fasta format). Sequence names correspond to codes described in Dataset S2 for flagella and NF-T3SS, and to Genbank accession numbers for F-/V- ATPases.
(TXT)

Dataset S2 List of systems analyzed along with taxonomic information, and equivalence for system codes found in Figure S5 and Dataset S1.
(XLS)

Figure S1 Learning dataset for discrimination between NF-T3SS and flagellar proteins based on Hmmer profile scores. For the subset of T3SS proteins used as a training dataset for the linear discriminant analysis shown in Figure 2 we show their protein profile scores using the NF-T3SS-specific profiles (X axis) and the flagellum-specific profiles (Y axis). Color codes indicate the different gene families. Filled circles indicate flagellar system proteins whereas open squares indicate NF-T3SS proteins.
(PDF)

Figure S2 Representation of each scenario for T3SS evolution in the phylogenetic analysis of the F-/V- ATPase family. The number of bootstrap trees in agreement with each scenario is indicated in cells. These numbers are shown for both the dataset used for the tree on Figure 3B (out of 997 bootstrap trees) and for a wider dataset including all curated systems (out of 974 trees), after the mark “&” (Text S1).
(PDF)

Figure S3 Individual phylogenies of the NF-T3SS core proteins with flagellar homologs. These trees were obtained with NF-T3SS proteins, and did not include flagellar proteins. (A) The eight collapsed individual trees. Branches with low support value (bootstrap<80%) were collapsed. Supports for some critical relationships in the rooted trees can be found in Table S4, black arrows indicate position of the flagellum sequences in the “rooted” versions of these trees (not shown). (B) These trees are the same as the trees presented in panel A, except that branches with bootstrap values below 80% were not collapsed.
(PDF)

Figure S4 Maximum likelihood phylogeny of the NF-T3SS using the concatenation of the eight core genes with homologs in the flagellum. The eight individual trees are displayed in Figure S3. For more clarity, strain names were replaced by the corresponding species names when the systems were monophyletic. Names in red correspond to the model NF-T3SSs used to build the protein profiles of the NF-T3SS core genes. Bootstrap supports are all shown for relationships between families, but within families bootstrap supports are indicated only when below 80%. The “Gene content” panel indicates the presence (black for clear homologs, or brown if found in a distant or unrelated clade-specific family with weak or no sequence similarity) and absence (grey) of other conserved NF-T3SS genes (see Materials and Methods). When not found in the protein dataset with our Hmmer search or with our clustering method, traces of genes were sought by performing tBlastn searches on complete genomes. These hits could correspond either to pseudogenes, fast evolving sequences, or sequencing errors. If traces were retrieved, the gene appears with a salmon box. The “Ecology” panel indicates bacterium/eukaryote associations. Large monophyletic clades correspond to the NF-T3SS families (colored boxes). “Chlamy” stands for Chlamydiales, “Desulfo” for Desulfovibrionales, “Myxo” for Myxococcales, “Rhizo” for Rhizobiales, and “Bups” for *Burkholderia pseudomallei* group. The black arrow indicates the position of the flagellum sequences in the “rooted” version of this tree (Figure S5), and the scale of the tree is given in substitutions per site.
(PDF)

Figure S5 Concatenate phylogeny of flagellar and non-flagellar T3SSs based on SctNJQRSTUV and their respective flagellar homologs (see Tables S1 or S4 for gene names). Branch supports correspond to the occurence of the splits in 100 rapid bootstrap trees. Color boxes surround the different NF-T3SS families. Yellow for Myxo, brown for Chlamy, red for Hrp2, orange for Ysc, green for Rhizo, cyan for Hrp1, violet for SPI2, and dark purple for SPI1. Two levels of taxonomy are indicated on the right. See Dataset S2 for equivalence between system codes and replicon names.
(PDF)

Protocol S1 Analysis of the F-/V- ATPase family.
(DOC)

Table S1 Nomenclature of NF-T3SS and flagellar components in various systems.
(DOC)

Table S2 List and justification of excluded NF-T3SS systems.
(DOC)

Table S3 List of model genomes used to build flagellar protein families and profiles.
(DOC)

Table S4 Support values (percentage of rapid bootstraps) for critical relationships in rooted phylogenies of T3SS genes.
(DOC)

Table S5 List of model genomes used to build NF-T3SS protein families and profiles (in red on Figure S4).
(DOC)

Text S1 Analysis of the F-/V- ATPase trees.
(DOC)

Text S2 The phylogeny of NF-T3SS.
(DOC)

Text S3 Checking phylogenetic signal consistency with Prunier.
(DOC)

Text S4 Tracing the origins of Rhizo secretins.
(DOC)
