# Supplementary figures and images for: The Non-Flagellar Type III Secretion System Evolved from the Bacterial Flagellum and Diversified into Host-Cell Adapted Systems
Source: PLoS Genet. 2012 Sep 27;8(9):e1002983. doi: 10.1371/journal.pgen.1002983 (PMC3459982; doi:10.1371/journal.pgen.1002983)

Flagellum profile scores

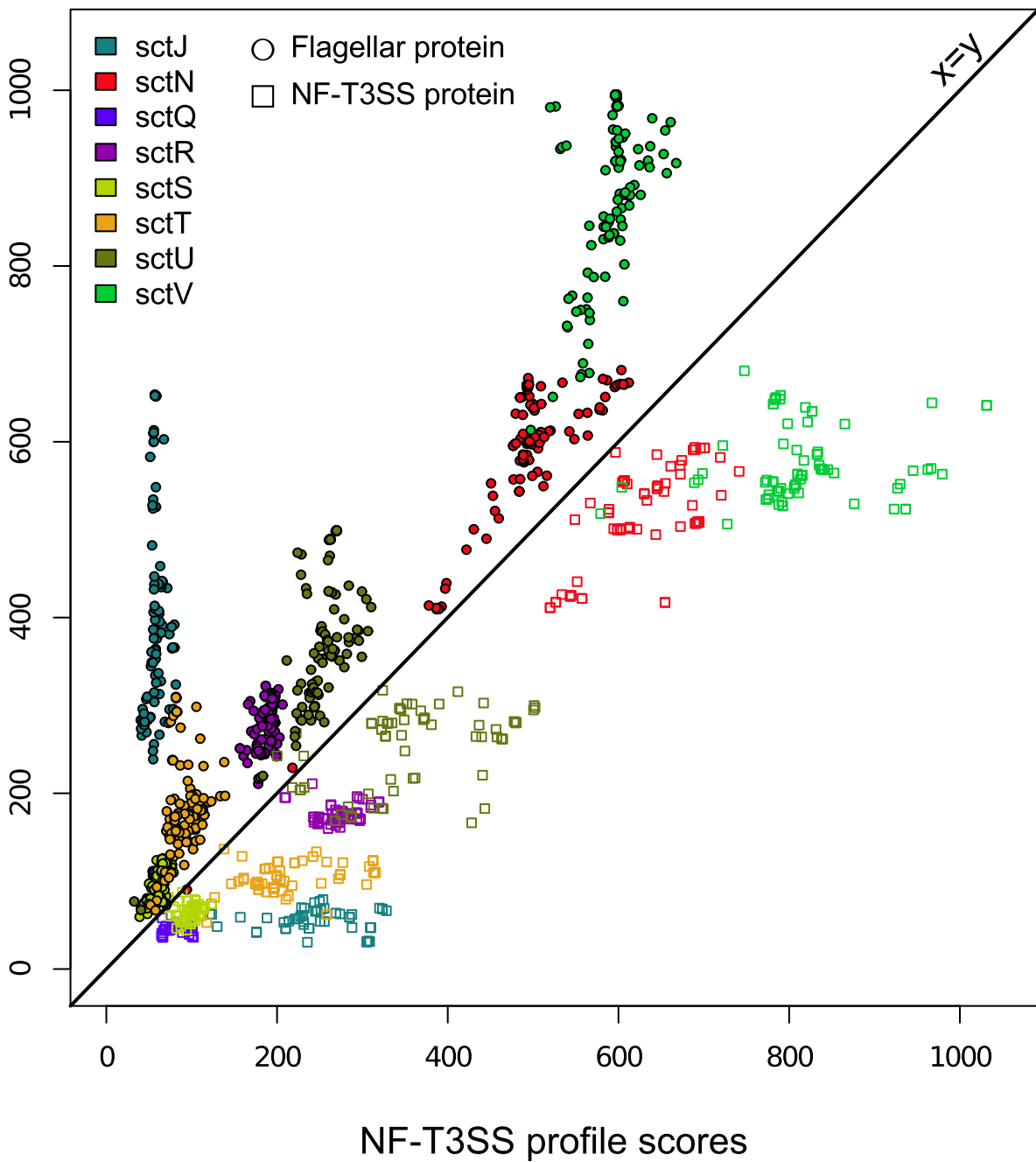

Supplement: Figure S1 — Learning dataset for discrimination between NF-T3SS and flagellar proteins based on Hmmer profile scores. For the subset of T3SS proteins used as a training dataset for the linear discriminant analysis shown in Figure 2 we show their protein profile scores using the NF-T3SS-specific profiles (X axis) and the flagellum-specific profiles (Y axis). Color codes indicate the different gene families. Filled circles indicate flagellar system proteins whereas open squares indicate NF-T3SS proteins. (PDF) [file pgen.1002983.s003.pdf]

A

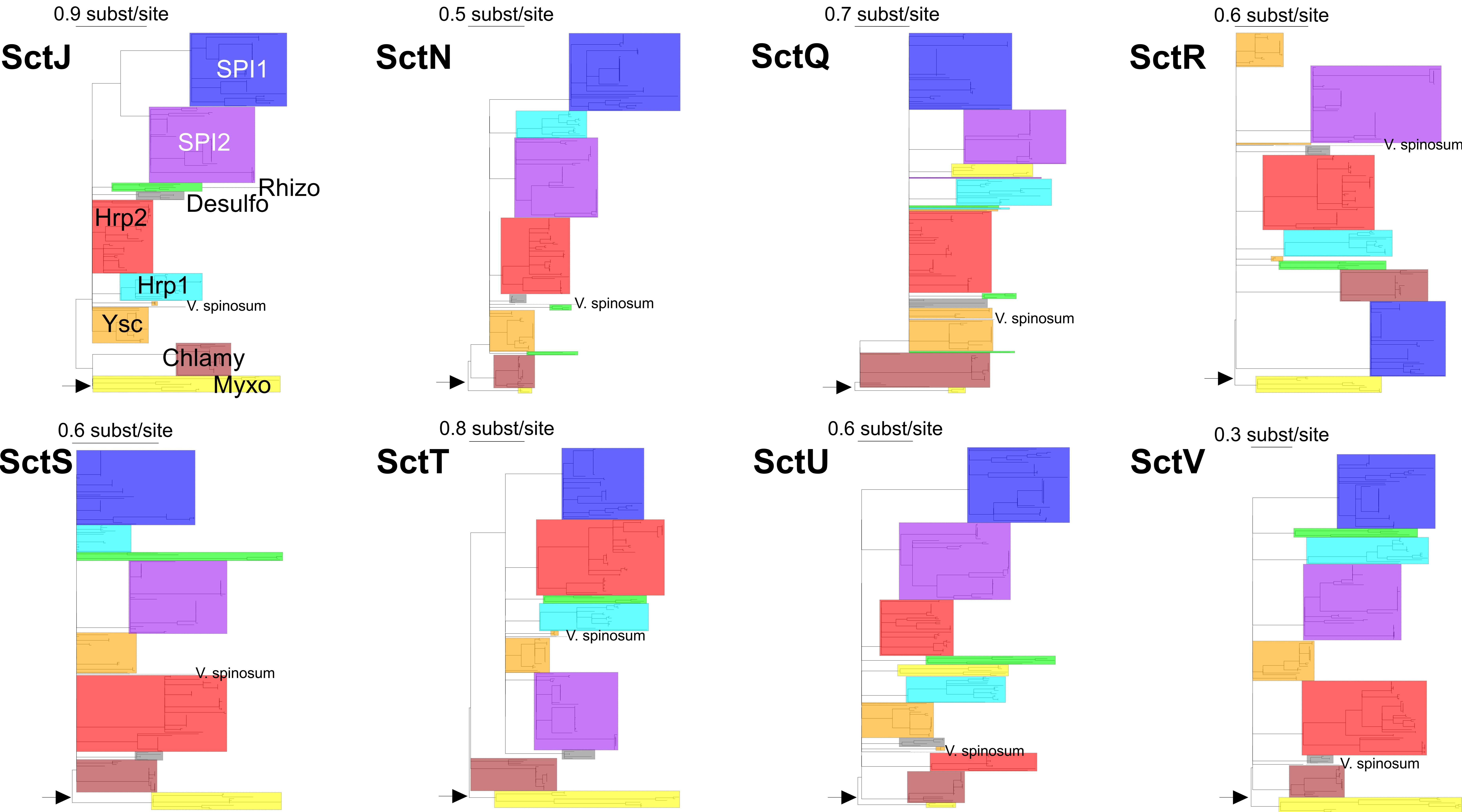

B

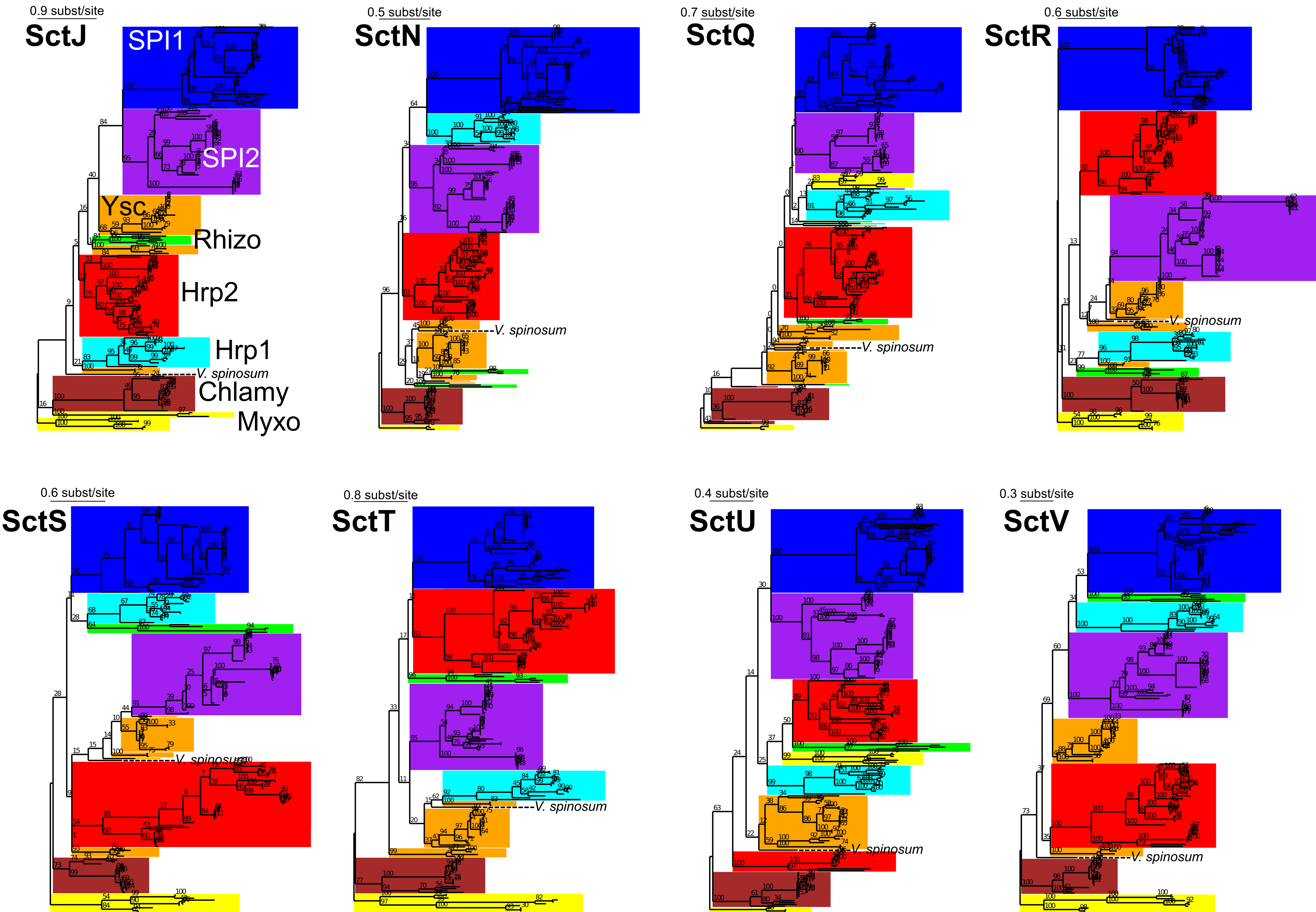

Supplement: Figure S3 — Individual phylogenies of the NF-T3SS core proteins with flagellar homologs. These trees were obtained with NF-T3SS proteins, and did not include flagellar proteins. (A) The eight collapsed individual trees. Branches with low support value (bootstrap<80%) were collapsed. Supports for some critical relationships in the rooted trees can be found in Table S4, black arrows indicate position of the flagellum sequences in the “rooted” versions of these trees (not shown). (B) These trees are the same as the trees presented in panel A, except that branches with bootstrap values below 80% were not collapsed. (PDF) [file pgen.1002983.s005.pdf]

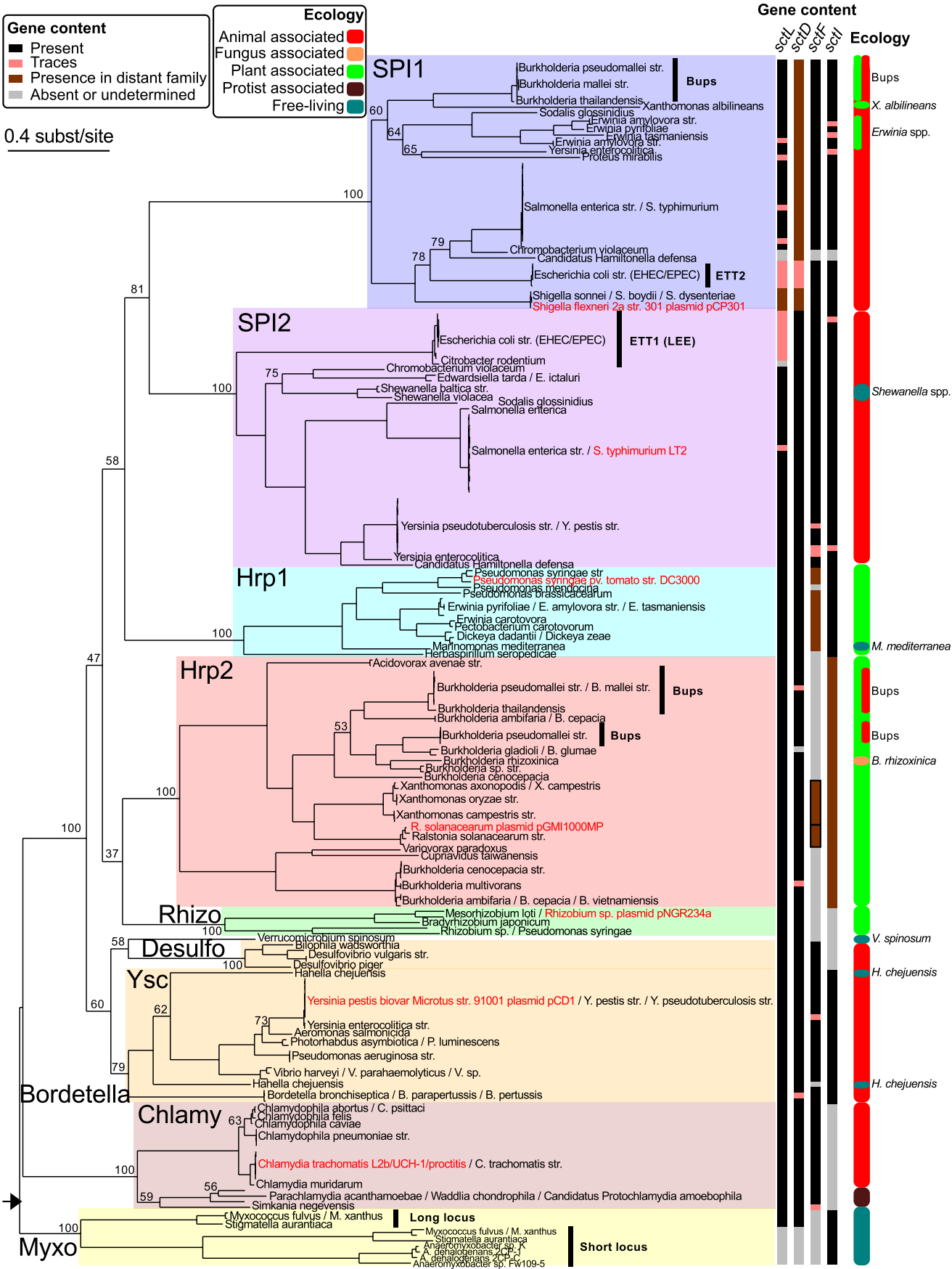

Supplement: Figure S4 — Maximum likelihood phylogeny of the NF-T3SS using the concatenation of the eight core genes with homologs in the flagellum. The eight individual trees are displayed in Figure S3. For more clarity, strain names were replaced by the corresponding species names when the systems were monophyletic. Names in red correspond to the model NF-T3SSs used to build the protein profiles of the NF-T3SS core genes. Bootstrap supports are all shown for relationships between families, but within families bootstrap supports are indicated only when below 80%. The “Gene content” panel indicates the presence (black for clear homologs, or brown if found in a distant or unrelated clade-specific family with weak or no sequence similarity) and absence (grey) of other conserved NF-T3SS genes (see Materials and Methods). When not found in the protein dataset with our Hmmer search or with our clustering method, traces of genes were sought by performing tBlastn searches on complete genomes. These hits could correspond either to pseudogenes, fast evolving sequences, or sequencing errors. If traces were retrieved, the gene appears with a salmon box. The “Ecology” panel indicates bacterium/eukaryote associations. Large monophyletic clades correspond to the NF-T3SS families (colored boxes). “Chlamy” stands for Chlamydiales, “Desulfo” for Desulfovibrionales, “Myxo” for Myxococcales, “Rhizo” for Rhizobiales, and “Bups” for Burkholderia pseudomallei group. The black arrow indicates the position of the flagellum sequences in the “rooted” version of this tree (Figure S5), and the scale of the tree is given in substitutions per site. (PDF) [file pgen.1002983.s006.pdf]

0.5 subst/site

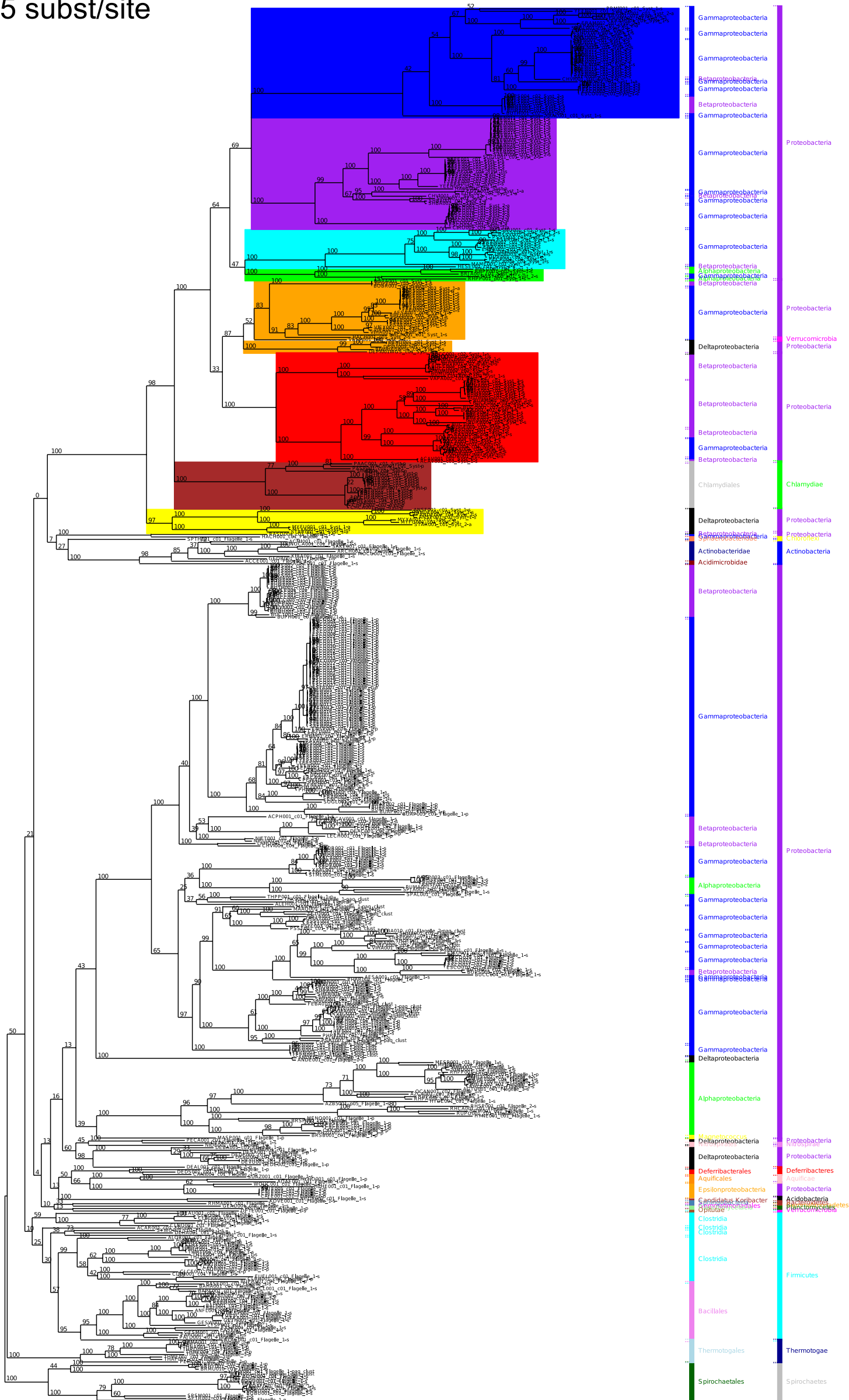

NF-T3SS

Flagella

Supplement: Figure S5 — Concatenate phylogeny of flagellar and non-flagellar T3SSs based on SctNJQRSTUV and their respective flagellar homologs (see Tables S1 or S4 for gene names). Branch supports correspond to the occurence of the splits in 100 rapid bootstrap trees. Color boxes surround the different NF-T3SS families. Yellow for Myxo, brown for Chlamy, red for Hrp2, orange for Ysc, green for Rhizo, cyan for Hrp1, violet for SPI2, and dark purple for SPI1. Two levels of taxonomy are indicated on the right. See Dataset S2 for equivalence between system codes and replicon names. (PDF) [file pgen.1002983.s007.pdf]
