# Supplementary material for: The Non-Flagellar Type III Secretion System Evolved from the Bacterial Flagellum and Diversified into Host-Cell Adapted Systems
Source: PLoS Genet. 2012 Sep 27;8(9):e1002983. doi: 10.1371/journal.pgen.1002983 (PMC3459982; doi:10.1371/journal.pgen.1002983)
Supplement: Figure S2 — Representation of each scenario for T3SS evolution in the phylogenetic analysis of the F-/V- ATPase family. The number of bootstrap trees in agreement with each scenario is indicated in cells. These numbers are shown for both the dataset used for the tree on Figure 3B (out of 997 bootstrap trees) and for a wider dataset including all curated systems (out of 974 trees), after the mark “&” (Text S1). (PDF) [file pgen.1002983.s004.pdf]

	Flagella and/or NF-T3SSs monophyletic	Flagella and NF-T3SSs polyphyletic	Total
Early split	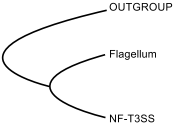<br>20 & 10	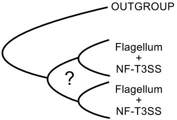<br>51 & 5	7.1% & 1.5%
NF-T3SS 1st	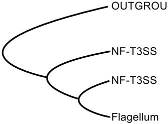<br>92 & 187	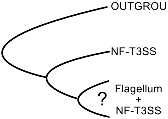<br>0 & 70	9.2% & 26.4%
Flagellum 1st	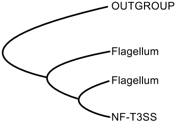<br>752 & 435	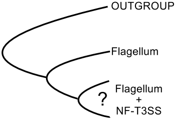<br>82 & 267	83.7% & 72.1%
