# Supplementary material for: The Non-Flagellar Type III Secretion System Evolved from the Bacterial Flagellum and Diversified into Host-Cell Adapted Systems
Source: PLoS Genet. 2012 Sep 27;8(9):e1002983. doi: 10.1371/journal.pgen.1002983 (PMC3459982; doi:10.1371/journal.pgen.1002983)
Supplement: Protocol S1 — Analysis of the F-/V- ATPase family. (DOC) [file pgen.1002983.s008.doc]

# Protocol S1. Analysis of the F-/V- ATPase family.

### Extraction and selection of sequences for the F-/V- ATPase family.

We extracted sequences of the T3SS outgroup VATA, VATB, ATPA and ATPB families from Genbank using accession numbers found in . We built an alignment (Muscle , default parameters) and a tree (RAxML , LG+G8+F ) with all these sequences, and used Bpp-Physamp from the Bio++ Program suite, (http://home.gna.org/bppsuite/) in order to select the 100 more phylogenetically distinct and longer sequences from those retrieved (deletion_method=sample, choice_criterion=length.complete).

### Analysis of the F-/V- ATPase trees: methods

We wrote a Bio++ program to count the different possible evolutionary scenarios in F-/V- ATPase bootstrap trees. This program was used to test the monophyly of the outgroup sequences, flagellar sequences, and NF-T3SS sequences. We first excluded trees with non monophyletic outgroup sequences. For remaining trees for which neither the flagellar, nor the NF-T3SS sequences were monophyletic, we computed the branch position of the last common ancestors (LCA) of the flagellar and of the NF-T3SS sequences, and then calculated a distance corresponding to the number of nodes separating the LCA of each system to the branch leading to the outgroup.

# References

1. Mulkidjanian AY, Makarova KS, Galperin MY, Koonin EV (2007) Inventing the dynamo machine: the evolution of the F-type and V-type ATPases. Nature reviews Microbiology 5: 892-899.

2. Edgar RC (2004) MUSCLE: multiple sequence alignment with high accuracy and high throughput. Nucleic Acids Res 32: 1792-1797.

3. Stamatakis A (2006) RAxML-VI-HPC: maximum likelihood-based phylogenetic analyses with thousands of taxa and mixed models. Bioinformatics 22: 2688-2690.

4. Le SQ, Gascuel O (2008) An improved general amino acid replacement matrix. Mol Biol Evol 25: 1307-1320.

5. Dutheil J, Gaillard S, Bazin E, Glemin S, Ranwez V, et al. (2006) Bio++: a set of C++ libraries for sequence analysis, phylogenetics, molecular evolution and population genetics. BMC Bioinformatics 7: 188.
