# Supplementary material for: The Non-Flagellar Type III Secretion System Evolved from the Bacterial Flagellum and Diversified into Host-Cell Adapted Systems
Source: PLoS Genet. 2012 Sep 27;8(9):e1002983. doi: 10.1371/journal.pgen.1002983 (PMC3459982; doi:10.1371/journal.pgen.1002983)
Supplement: Table S1 — Nomenclature of NF-T3SS and flagellar components in various systems. (DOC) [file pgen.1002983.s009.doc]

# Table S1. Nomenclature of NF-T3SS and flagellar components in various systems.

| **NF-T3SS** | **Flagellum** | **Yersinia**  **Ysc** | **Salmonella SPI1** | **Shigella**  **SPI1** | **B. pseudomallei**  **SPI1** | **E. coli LEE SPI2** | **P. syringae**  **Hrp1** | **R. solanacea-rum**  **Hrp2** | **X. cam-pestris**  **Hrp2** | **Chlamydia** | **Rhizobium OLS** | **Rhizobium** | **Function** |
| --- | --- | --- | --- | --- | --- | --- | --- | --- | --- | --- | --- | --- | --- |
| **SctW** | -- | YopN | InvE | MxiC | BsaP | SepL | HrpJ | - | - | CopN | - | - | Regulator translocation/probably cytoplasmic/gatekeeper |
| **SctN*** | FliI | YscN | InvC | Spa47 | BsaS | EscN | HrpJ4 | HrpE | HrpB6 | CdsN | HrcN;Y4yI | RhcN | ATPase |
| **SctR*** | FliP | YscR | SpaP | Spa24 | BsaW | EscR | HrpW | HrpT | ORF2 | CdsR | HrcR;Y4yL | RhcR | Type 3 secretion apparatus |
| **SctS*** | FliQ | YscS | SpaQ | Spa9 | BsaX | EscS | HrpO | HrpU | HrcS | CdsS | HrcS;Y4yM | RhcS | Type 3 secretion apparatus |
| **SctT*** | FliR | YscT | SpaR | Spa29 | BsaY | EscT | HrpX | HrpC | HrpB8 | CdsT | HrcT;Y4yN | RhcT | Type 3 secretion apparatus |
| **SctU*** | FlhB | YscU | SpaS | Spa40 | BsaZ | EscU | HrpY | HrpN | HrcC | CdsU | HrcU;Y4yO | RhcU | Type 3 secretion apparatus/ substrate switching |
| **SctV*** | FlhA | YscV/LcrD | InvA | MxiA | BsaQ | EscV | HrpI | HrpO | HrpC2 | CdsV | HrcV;Y4yR | RhcV | Type 3 secretion apparatus |
| **SctQ*** | FliN/FliM | YscQ | SpaO | Spa33 | BsaV | EscQ | HrpU | HrpQ | HrcQ | CdsQ | HrcQ | RhcQ | Sorting platform |
| **SctL°** | FliH | YscL | OrgB | MxiN | OrgB | EscL | HrpE | HrpF | HrpB5 | CdsL | NolV | RhcL | Sorting platform |
| **SctK** | - | YscK | OrgA | MxiK | OrgA | ORF4 | HrpD | - | - | - | - | - | Sorting platform |
| **SctJ*** | FliF | YscJ | PrgK | MxiJ | BsaJ | EscJ | HrpC | HrpI | HrpB3 | CdsJ | NolT | RhcJ | Smaller inner ring protein |
| **SctD°** | -- | YscD | PrgH | MxiG | BsaM | EscD | HrpQ | HrpW | HrpD5 | CdsD | Y4yQ | - | Larger inner ring protein |
| **SctC*** | -- | YscC | InvG | MxiD | BsaO | EscC | HrpH | HrpA | HrpA1 | CdsC | Y4yJ/NolW | RhcC1/RhcC2 | Secretin/OM ring |
|  | -- | YscW | InvH | MxiM | - | - | - | - | - | - | - | - | Pilotin, assists secretin insertion into OM |
| **-** | FlgH | - | - | - | - | - | - | - | - | - | - | - | Flagellum outer membrane ring lipoprotein |
| **SctF°^†^** | FliC/FlgL^†^ | YscF | PrgI | MxiH | BsaL | EscF | - | - | - | CdsF |  | - | Needle filament |
| **SctI°** | -- | YscI | PrgJ | MxiI | BsaK | EscI | HrpB | HrpJ | HrpB2 | - | - | - | Inner rod protein |
| **SctP** | FliK | YscP | InvJ | Spa32 | BsaU | ORF16 | HrpP | HpaP | HpaC | - | - | - | Needle length determinant/substrate switching/regulator/gatekeeper/extracellular |
| **-** | - | YopD | SipC | IpaC | BipC | EspB | - | - | - | - | - | - | Translocon |
| **-** | - | YopB | SipB | IpaB | BipB | EspD | HrpK1 | - | HrpF | CopB2 | - | - | Translocon |
| **-** | - | LcrV | SipD | IpaD | BipD | EspA | - | - | - | CT584 | - | - | Needle tip |
| **SctO** | - | YscO | InvI | Spa13 | BsaT | - | HrpO | - | - | CdsO/MscS | ORF7 | - | Chaperone escort protein; essential for export of needle subunits |
| **-** | - | - | - | - | - | - | HrpA | - | - | - | - | - | Pilus |
| **-** | - | - | - | - | - | - | - | HrpY | HrpE | - | - | - | Pilus |
| **--** | FlgB*C*FG | - | - | - | - | - | - | - | - | - | - | - | Rod/transmission shaft |
| **--** | FlgK | - | - | - | - | - | - | - | - | - | - | - | Hook associated protein |
| **--** | FlgE | - | - | - | - | - | - | - | - | - | - | - | Hook universal joint |
| **--** | FliE* | - | - | - | - | - | - | - | - | - | - | - | Rod/Basal body |

* gene used in profiles for system detection ; ° non ubiquitous gene in the system ; ^†^ homology was previously proposed based on structural analogies, however our profiles could retrieve a sequence similarity supporting this homology; -- no homolog could be detected based on sequence analysis; - missing information or no homolog. Gene names separated by a "/" indicate that 2 homologs were found, and when separated by ";", that alternate names were found.
