# Supplementary material for: The Non-Flagellar Type III Secretion System Evolved from the Bacterial Flagellum and Diversified into Host-Cell Adapted Systems
Source: PLoS Genet. 2012 Sep 27;8(9):e1002983. doi: 10.1371/journal.pgen.1002983 (PMC3459982; doi:10.1371/journal.pgen.1002983)
Supplement: Table S2 — List and justification of excluded NF-T3SS systems. (DOC) [file pgen.1002983.s010.doc]

# Table S2. List and justification of excluded NF-T3SS systems.

| **Replicon** | **Diagnostic** |
| --- | --- |
| *Agrobacterium radiobacter* K84, chr. 2 | Long branches, missing secretin, shorter matches for *sctJ, sctS, sctU.* Probably degraded. |
| *Burkholderia phytofirmans* PsJN, chr. 2 | Long branches, shorter match for *sctU.* Probably degraded. |
| *Burkholderia xenovorans* LB400, chr. 1 | Long branches, *sctS* missing, shorter match for *sctJ.* Probably degraded. |
| *Lawsonia intracellularis* PHE/MN1-0 | Ambiguous cluster. Groups with Desulfo NF-T3SS family from ATPase phylogeny . |
| *Mesorhizobium* sp. BNC1 | Long branches |
| *Photorhabdus asymbiotica* subsp. asymbiotica ATCC 43949 | Long branches, *sctJ* and *sctT* missing. Shorter matches for *sctN, sctR, sctV*. Probably degraded. Putative system described in . |
| *Vibrio parahaemolyticus* RIMD 2210633, chr. II | Long branches, *sctT* missing, shorter match for *sctV.* Branches near from Ysc V. parahaemolyticus systems in multiple gene phylogenies . |

Chromosome (chr.) numbers are mentioned only when more than one chromosome is present.

# References

1. Sait M, Kamneva OK, Fay DS, Kirienko NV, Polek J, et al. (2011) Genomic and Experimental Evidence Suggests that Verrucomicrobium spinosum Interacts with Eukaryotes. Front Microbiol 2: 211.

2. Stackebrandt E (2006) Taxonomic parameters revisited: tarnished gold standards. Microb Today 8: 152-155.

3. Okada N, Iida T, Park KS, Goto N, Yasunaga T, et al. (2009) Identification and characterization of a novel type III secretion system in trh-positive Vibrio parahaemolyticus strain TH3996 reveal genetic lineage and diversity of pathogenic machinery beyond the species level. Infect Immun 77: 904-913.
