# Supplementary material for: The Non-Flagellar Type III Secretion System Evolved from the Bacterial Flagellum and Diversified into Host-Cell Adapted Systems
Source: PLoS Genet. 2012 Sep 27;8(9):e1002983. doi: 10.1371/journal.pgen.1002983 (PMC3459982; doi:10.1371/journal.pgen.1002983)
Supplement: Table S3 — List of model genomes used to build flagellar protein families and profiles. (DOC) [file pgen.1002983.s011.doc]

# Table S3. List of model genomes used to build flagellar protein families and profiles.

| **Clade** | **Model** |
| --- | --- |
| **Proteobacteria** | *Escherichia coli* str. K-12 substr. MG1655 |
| **Bacteroidetes-chlorobi** | *Salinibacter ruber* DSM 13855 |
| **Spirochaetes** | *Borrelia burgdorferi* B31 |
| **Bacillales** | *Bacillus subtilis* subsp. subtilis str. 168 |
| **Actinobacteria** | *Acidothermus cellulolyticus* 11B |
| **Acidobacteria** | *Acidobacterium capsulatum* ATCC 51196 |
| **Aquificae** | *Sulfurihydrogenibium* sp. YO3AOP1 |
| **Clostridia** | *Clostridium difficile* 630 |
