# Supplementary material for: The Non-Flagellar Type III Secretion System Evolved from the Bacterial Flagellum and Diversified into Host-Cell Adapted Systems
Source: PLoS Genet. 2012 Sep 27;8(9):e1002983. doi: 10.1371/journal.pgen.1002983 (PMC3459982; doi:10.1371/journal.pgen.1002983)
Supplement: Table S4 — Support values (percentage of rapid bootstraps) for critical relationships in rooted phylogenies of T3SS genes. (DOC) [file pgen.1002983.s012.doc]

# Table S4. Support values (percentage of rapid bootstraps) for critical relationships in rooted phylogenies of T3SS genes.

| **Genes**  **(NF-T3SS/ flagellum)** | **Sites selected in alignment** | **Flagellum /**  **NF-T3SS split** | **Myxo NF-T3SS / other NF-T3SS split** | **Chlamy NF-T3SS / other NF-T3SS split** |
| --- | --- | --- | --- | --- |
| sctJ / fliF | 136 | 100 | 30 | 14 |
| sctN / fliI | 334 | 94 | 78 | 87 |
| sctQ / fliN-fliM | 61 | 26 | -- | 33 |
| sctR / fliP | 169 | 100 | 83 | 48 |
| sctS / fliQ | 72 | 72 | 93 | -- |
| sctT / fliR | 157 | 95 | 39 | 87 |
| sctU / flhB | 240 | 47* | 20 | -- |
| sctV / flhA | 489 | 100 | 63 | 59 |

“--” indicates that this pattern was not retrieved in the phylogeny.

* In sctU/flhB phylogeny, two flagellar sequences branched among the Myxo NF-T3SS clade (60% of rapid bootstrap support).
