# Supplementary material for: The Non-Flagellar Type III Secretion System Evolved from the Bacterial Flagellum and Diversified into Host-Cell Adapted Systems
Source: PLoS Genet. 2012 Sep 27;8(9):e1002983. doi: 10.1371/journal.pgen.1002983 (PMC3459982; doi:10.1371/journal.pgen.1002983)
Supplement: Table S5 — List of model genomes used to build NF-T3SS protein families and profiles (in red on Figure S4). (DOC) [file pgen.1002983.s013.doc]

# Table S5. List of model genomes used to build NF-T3SS protein families and profiles (in red on Fig. S4).

| **System** | **Model** |
| --- | --- |
| **Ysc** | *Yersinia pestis* biovar Microtus str. 91001 plasmid pCD1 |
| **SPI1** | *Shigella flexneri* 2a str. 301 plasmid pCP301 |
| **SPI2** | *Salmonella typhimurium* LT2 |
| **Hrp1** | *Pseudomonas syringae* pv. tomato str. DC3000 |
| **Hrp2** | *Ralstonia solanacearum* GMI1000 plasmid pGMI1000MP |
| **Rhizobiales** | *Rhizobium* sp. NGR234 plasmid pNGR234a |
| **Chlamydiae** | *Chlamydia trachomatis* L2b/UCH-1/proctitis |
