# Supplementary material for: The Non-Flagellar Type III Secretion System Evolved from the Bacterial Flagellum and Diversified into Host-Cell Adapted Systems
Source: PLoS Genet. 2012 Sep 27;8(9):e1002983. doi: 10.1371/journal.pgen.1002983 (PMC3459982; doi:10.1371/journal.pgen.1002983)
Supplement: Text S1 — Analysis of the F-/V- ATPase trees. (DOC) [file pgen.1002983.s014.doc]

# Text S1. Analysis of the F-/V- ATPase trees.

In addition to the maximum likelihood tree we built for the F-/V- ATPase (Fig. 3) and to further assess the relative probability of each of the three possible scenarios for the evolution of NF-T3SS and flagella (early split between flagellar and non-flagellar systems, NF-T3SS emerging first, and flagellum emerging first), we counted different occurrences of these scenarios (see Protocol S1) in 1000 bootstrap tree replicates built from two F-/V- ATPase sequence alignments: the one whose tree is presented in Fig. 3B (“sub”) (alignment in Dataset S1), and the alignment of a wider dataset (“wd”), which included all curated systems. We removed the cases where the outgroup sequences were not monophyletic (3 in “sub”, 26 in “wd”) and divided the resulting trees into six scenarios. First we considered trees where either the NF-T3SS or the flagellum or both were monophyletic (864 in “sub”, 632 trees in “wd”, first column in Fig. S2). This shows that only 7.1% (in “sub”) and 1.5% (in “wd”) of these bootstrap trees fit the scenario of an early split between the NF-T3SS and the flagellum. The scenario of a root within flagellum, with NF-T3SS appearing as a derived state is preponderant over the scenario where the flagellum is derived from the NF-T3SS (87% versus 10.6% of the 864 “sub” bootstrap trees and 68.8% versus 29.6% of the 632 “wd” bootstrap trees). This is in agreement with the most likely trees we reconstructed for the two sets.

We also analyzed the trees where both NF-T3SS and flagellum were polyphyletic: 342 in “wd” and 133 in “sub” (second column in Fig. S2). Phylogenetic uncertainty sometimes artifactually misplaces a few fast evolving taxa of one clade within others, thereby disrupting monophyly and although this scenario is incorrect in the respect of a single origin for the flagellum and a single origin for the NF-T3SS, it can still bring information to our key question that is which system emerged first (Fig. S2). For the “wd” dataset, we computed for each of the 342 “polyphyletic” trees the distance to the outgroup of the last common ancestors (LCA) of the NF-T3SS and of the flagellum (see Protocol S1). This shows that the flagellum-first scenario is the most frequently found since the distance to the outgroup was significantly higher for the NF-T3SS LCA than for the flagellum LCA (p-value< 0.0001, Wilcoxon test). Overall, the analysis of the non-parametric bootstrap trees from the set of selected sequences and from the wider dataset including all curated systems are in line with the most likely trees favoring the flagellum first scenario in 72.1% of the cases for the whole dataset, and in 83.7% of the cases in the dataset with fewer sequences. The NF-T3SS first scenario is favored in 26.4% of the cases in the wide dataset and only 9.2% in the “sub” dataset. The early split scenario is only observed in 1.5% of “wd” and 7.1% of “sub” datasets.
