# Supplementary material for: The Non-Flagellar Type III Secretion System Evolved from the Bacterial Flagellum and Diversified into Host-Cell Adapted Systems
Source: PLoS Genet. 2012 Sep 27;8(9):e1002983. doi: 10.1371/journal.pgen.1002983 (PMC3459982; doi:10.1371/journal.pgen.1002983)
Supplement: Text S2 — The phylogeny of NF-T3SS. (DOC) [file pgen.1002983.s015.doc]

# Text S2. The phylogeny of NF-T3SS.

After the split with Chlamydiales, the secretin-encoding NF-T3SS diversified very rapidly into a series of types in Proteobacteria (one is also found in Verrucomicrobia, see below) (Fig. 4, Fig. S4). These monophyletic types are all very well supported in the tree (100% bootstrap except for the Ysc system). While our data reinforces most elements of the present classification of NF-T3SSs , it also brings some new points to the fore. Desulfo (for Desulfovibrionales, a Delta-proteobacterial clade), which encompasses both commensals and pathogens, form a highly supported independent clade, possibly related to the Ysc system. Second, even if not highly supported in our multi-protein phylogeny (58% bootstrap, Figs. 4 and S4), *V. spinosum* NF-T3SS is positioned within proteobacterial NF-T3SSs, and apart from Chlamydiales systems. This was previously proposed from a phylogenetic tree of the T3SS ATPase , and suggests the spread of NF-T3SS from Proteobacteria to other clades of diderms by horizontal gene transfer. Third, the Ysc type, previously proposed to include *Bordetella* systems , has a weaker support than the other types (79% of bootstrap in the concatenate tree) and shows a clear early split between the *Bordetella* and the remaining Ysc systems. These two sub-clades are both very well supported (100% and 99% of bootstrap respectively) suggesting that *Bordetella* might be better classed apart from the Ysc, which is also supported by the paucity of shared derived traits between Ysc and *Bordetella* systems . Fourth, we observe within the group of Rhizobiales one Gamma-proteobacterium (*P. syringae*) with a NF-T3SS whose function is unknown , and that is not the typical Hrp1 *P. syringae* system.

# References

1. Troisfontaines P, Cornelis GR (2005) Type III secretion: more systems than you think. Physiology 20: 326-339.

2. Sait M, Kamneva OK, Fay DS, Kirienko NV, Polek J, et al. (2011) Genomic and Experimental Evidence Suggests that Verrucomicrobium spinosum Interacts with Eukaryotes. Front Microbiol 2: 211.

3. Foultier B, Troisfontaines P, Muller S, Opperdoes FR, Cornelis GR (2002) Characterization of the ysa pathogenicity locus in the chromosome of Yersinia enterocolitica and phylogeny analysis of type III secretion systems. J Mol Evol 55: 37-51.

4. Gophna U, Ron EZ, Graur D (2003) Bacterial type III secretion systems are ancient and evolved by multiple horizontal-transfer events. Gene 312: 151-163.

5. Pallen MJ, Beatson SA, Bailey CM (2005) Bioinformatics, genomics and evolution of non-flagellar type-III secretion systems: a Darwinian perspective. FEMS Microbiol Rev 29: 201-229.

6. Lindeberg M, Myers CR, Collmer A, Schneider DJ (2008) Roadmap to new virulence determinants in Pseudomonas syringae: insights from comparative genomics and genome organization. Mol Plant Microbe Interact 21: 685-700.
