# Supplementary material for: The Non-Flagellar Type III Secretion System Evolved from the Bacterial Flagellum and Diversified into Host-Cell Adapted Systems
Source: PLoS Genet. 2012 Sep 27;8(9):e1002983. doi: 10.1371/journal.pgen.1002983 (PMC3459982; doi:10.1371/journal.pgen.1002983)
Supplement: Text S3 — Checking phylogenetic signal consistency with Prunier. (DOC) [file pgen.1002983.s016.doc]

# Text S3. Checking phylogenetic signal consistency with Prunier.

In order to investigate putative inconsistencies between the individual gene histories and the phylogenomic tree built by concatenating them, we used the program Prunier . Prunier aims at pinpointing topological differences between two trees that correspond to branches with support values above a given threshold. For each individual gene family tree (*sctN, sctJ, sctQ, sctR, sctS, sctT, sctU, sctV*, available in Fig. S3), we ran Prunier using the concatenate tree as a reference (Figs. 4, S4) with a bootstrap threshold of 80%. For two genes, *sctN* and *sctS*, we found no significant differences. For the other genes, we found a few differences, respectively 1, 1, 3, 2, 3 and 2 for *sctJ, sctQ, sctR, sctT, sctU* and *sctV*. All these differences corresponded to local rearrangements between the gene tree and the reference tree (in most cases, the difference corresponded to a single nearest neighbor interchange), very often for very short branches among systems in strains of a same species. Prunier considers the “reference” concatenate tree as fully resolved even if some branches are not. Therefore, we looked at the support values of conflicting branches in the reference tree too. This showed that the number of topological conflicts between the two trees that corresponded to branches above 80% support values in the two trees could be restored to 1, 0, 2, 2, 0 and 0 for respectively *sctJ, sctQ, sctR, sctT, sctU* and *sctV*. Importantly, no conflict appeared to be highly supported, as no difference remained when setting the threshold at 90% of bootstrap support values in the two trees. This shows that the trees of the 8 core genes are essentially congruent and share a same history.

# Reference

1. Abby SS, Tannier E, Gouy M, Daubin V (2010) Detecting lateral gene transfers by statistical reconciliation of phylogenetic forests. BMC Bioinformatics 11: 324.
