# Supplementary material for: The Non-Flagellar Type III Secretion System Evolved from the Bacterial Flagellum and Diversified into Host-Cell Adapted Systems
Source: PLoS Genet. 2012 Sep 27;8(9):e1002983. doi: 10.1371/journal.pgen.1002983 (PMC3459982; doi:10.1371/journal.pgen.1002983)
Supplement: Text S4 — Tracing the origins of Rhizo secretins. (DOC) [file pgen.1002983.s017.doc]

**Text S4. Tracing the origins of Rhizo secretins.**

The two genes encoding the Rhizobiales (Rhizo) secretin are not contiguous, as would be expected if they had resulted from a recent gene fission event. The gene *rhcC1*, which corresponds to the N-terminal part of the secretin, is found within a conserved cluster of NF-T3SS core genes. *rhcC2*, which encodes the Tad-related secretin (main text, Fig. 6), varies in its position while remaining peripheral in the locus (Fig. 6C). The N-terminal part of secretins is small and evolves fast, precluding the inference of a reliable phylogeny with very distant homologs. To identify the origin of *rhcC1* we searched the most similar homologous N-terminal parts of secretins with Blast. In all of the 100 best Blast hits, and in over 95% of the total hits in genomes (805 from 201 unique sequences, e-value ≤ 10-3), RhcC1 matched the N-terminal regions of SctC secretins of almost all NF-T3SS excluding Chlamy and the RhcC1 hits themselves. Therefore, while the C-terminus of the Rhizo secretin is closely related to the Tad system secretins, the small gene coded within the other T3SS core genes matches the N-terminus of the NF-T3SS secretins except that of Chlamy secretins, which have an independent origin. This very strongly suggests that the Tad-related RhcC2 of Rhizobiales arose from a secondary acquisition of a secretin in an ancestral NF-T3SS already harboring a “NF-T3SS-like” secretin (shared by all except Chlamy systems), to form the ancestral hybrid Rhizo system.

We then analyzed in detail the structure of the two Rhizo secretins RhcC1 and RhcC2 using PsortB 3.2 and InterProScan . PsortB was able to identify a peptide signal in both proteins, but not InterProScan. The latter found in RhcC2 the “secretin” domain (PFAM PF00263), and one “pil” domain (for “Pilus formation protein N terminal region”, PFAM PF13629). We found a similar domain organization in the closely related RcpA protein of *Aggregatibacter actinomycetemcomitans* that is the secretin of a Tad system. An additional domain, the “BON” domain (bacterial OsmY and nodulation ), was detected in RhcC2 of a clade of 3 of the 5 Rhizo NF-T3SSs. This domain architecture, which comprises a pil domain, a BON domain and a secretin domain, is the same of the CpaC protein of the Tad locus of *Caulobacter crescentus* (Fig. 6B). Thus the protein domain organization of SctC (N-terminal) regions that were not included in the secretins phylogenetic analyses because of their poor taxonomic representation provides further evidence for the close relatedness of secretins RhcC2 with those of the Tad locus. The RhcC1 protein matched no domain in InterProScan, but the Blast search previously performed indicated that it matched the first “N-domain” (PFAM PF3958) detected in “NF-T3SS-like” secretins, a domain that is systematically present in one or multiple copies in secretins (Fig. 6B). In short, phylogeny (main text), sequence similarity searches and protein domain analyses suggest that the most likely evolutionary scenario for the acquisition of the secretin by NF-T3SS is an independent acquisition in Chlamy and in the other NF-T3SSs. The ancestral Rhizo system then acquired a second secretin, RhcC2, from a Tad system. This was followed by the partial gene deletion of the original secretin RhcC1. This prefigures a case of partial homologous gene replacement.

**References**

1. Yu NY, Wagner JR, Laird MR, Melli G, Rey S, et al. (2010) PSORTb 3.0: improved protein subcellular localization prediction with refined localization subcategories and predictive capabilities for all prokaryotes. Bioinformatics 26: 1608-1615.

2. Quevillon E, Silventoinen V, Pillai S, Harte N, Mulder N, et al. (2005) InterProScan: protein domains identifier. Nucleic Acids Res 33: W116-120.

3. Yeats C, Bateman A (2003) The BON domain: a putative membrane-binding domain. Trends Biochem Sci 28: 352-355.

4. Korotkov KV, Gonen T, Hol WGJ (2011) Secretins: dynamic channels for protein transport across membranes. Trends Biochem Sci 36: 433-443.
